# A model for Scc2p stimulation of cohesin's ATPase and its inhibition by acetylation of Smc3p

**DOI:** 10.1101/gad.350278.122

**Published:** 2023-04-01

**Authors:** Kevin Boardman, Siheng Xiang, Fiona Chatterjee, Udochi Mbonu, Vincent Guacci, Douglas Koshland

**Affiliations:** Department of Molecular and Cell Biology, University of California, Berkeley, Berkeley, California 94720, USA

**Keywords:** ATPase, acetylation, cohesin, cohesion, ECO1, ESCO1, NIPBL, SCC2, SMC

## Abstract

The evolutionarily conserved cohesin complex mediates sister chromatid cohesion and facilitates mitotic chromosome condensation, DNA repair, and transcription regulation. These biological functions require cohesin's two ATPases, formed by the Smc1p and Smc3p subunits. Cohesin's ATPase activity is stimulated by the Scc2p auxiliary factor. This stimulation is inhibited by Eco1p acetylation of Smc3p at an interface with Scc2p. It was unclear how cohesin's ATPase activity is stimulated by Scc2p or how acetylation inhibits Scc2p, given that the acetylation site is distal to cohesin's ATPase active sites. Here, we identify mutations in budding yeast that suppressed the in vivo defects caused by Smc3p acetyl-mimic and acetyl-defective mutations. We provide compelling evidence that Scc2p activation of cohesin ATPase depends on an interface between Scc2p and a region of Smc1p proximal to cohesin's Smc3p ATPase active site. Furthermore, substitutions at this interface increase or decrease ATPase activity to overcome ATPase modulation by acetyl-mimic and acetyl-null mutations. Using these observations and an existing cryo-EM structure, we propose a model for regulating cohesin ATPase activity. We suggest that Scc2p binding to Smc1p causes the adjacent Smc1p residues and ATP to shift, stimulating Smc3p's ATPase. This stimulatory shift is inhibited through acetylation of the distal Scc2p–Smc3p interface.

## Abstract (precis)

In this study, Boardman et al. describe how interactions between auxiliary factor Scc2p and cohesin components Smc1p and Smc3p are crucial for the complex's ATPase activity. They propose a model in which Scc2p–Smc1p binding stimulates Smc3p's ATPase by shifting ATP into the active site, while Smc3p acetylation interferes with the Scc2p–Smc1p interface and inhibits ATPase activity.

The evolutionarily conserved protein complex called cohesin mediates sister chromatid cohesion and facilitates mitotic chromosome condensation, DNA repair, and transcription regulation. Cohesin is thought to perform these remarkably diverse biological functions through the complex control of its two activities: tethering two chromatin regions together (within or between DNA molecules) or extruding chromatin loops. Elucidating the different mechanisms of cohesin regulation and their coordination remains an important but elusive goal. Here, our studies in *Saccharomyces cerevisiae* provide a molecular mechanism for the coordinated control of cohesin by two of its key regulators: Scc2p and Eco1p.

Cohesin's core complex contains four subunits, which in budding yeast are called Smc1p, Smc3p, Scc3p, and Mcd1p (Scc1p) (Fig. 1A). Cohesin has ATPase activity. This activity requires two active sites (Smc3 ATPase and Smc1 ATPase) that are formed through the heterodimerization of the Smc1p and Smc3p head domains (Supplemental Fig. S1A; Weitzer et al. 2003; Arumugam et al. 2003). Both active sites are required for cohesin's ATPase activity, its loading onto chromosomes, and all of its biological activities (Arumugam et al. 2003; Weitzer et al. 2003). In addition to the core complex, a heterodimer of Scc2p and Scc4p is required for cohesin to bind chromosomes and extrude loops (Davidson et al. 2019; Ciosk et al. 2000; Bauer et al. 2021). These activities are thought to derive from Scc2p's ability to stimulate cohesin's ATPase activity (Murayama and Uhlmann 2014; Çamdere et al. 2015; Petela et al. 2018). Recent cryo-EM structures of yeast and human cohesin with Scc2p and its human ortholog, NIPBL, show that Scc2p has multiple interactions with cohesin's head domains and Smc3p's coiled coil (Collier et al. 2020; Shi et al. 2020). The presence of these interfaces strongly suggests that Scc2p directly regulates the cohesin head domain and its ATPase activity.

**Figure 1.**
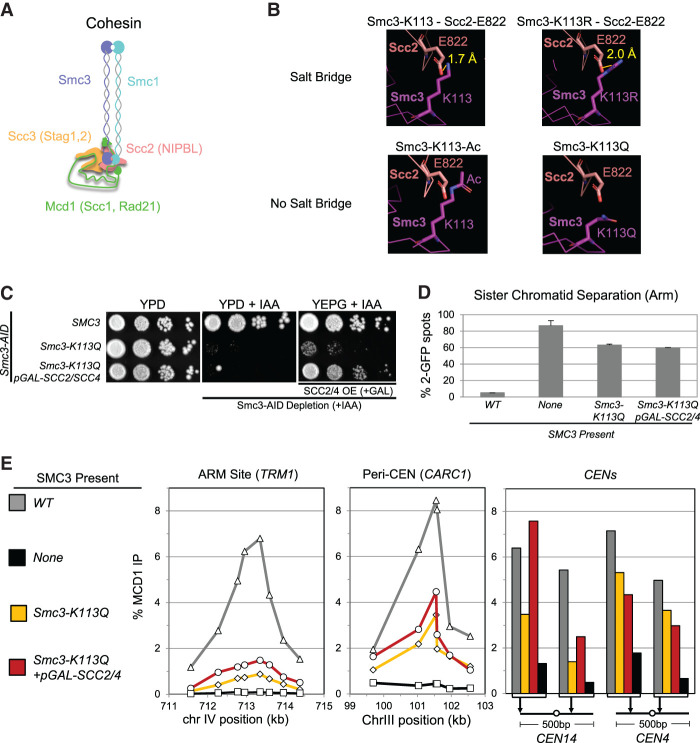
Characterization of the acetyl mimic and its suppression by Scc2p/Scc4p overexpression. (*A*) Cartoon depiction of cohesin (Smc1p, Smc3p, Scc3p, and Mcd1p) with the associated stimulatory factor Scc2p (NIPBL). (*B*) Cryo-EM structure of *S. cerevisiae* cohesin (PDB ID: 6ZZ6) (Collier et al. 2020) illustrating that Smc3p-K113 acetylation eliminates a salt bridge with Scc2p-E822. (Magenta) Smc3p-K113, (salmon) Scc2p-E822, (yellow) distance for a putative salt bridge. The *top* panels show that a salt bridge can form between Smc3p-K113 (*left*) or the K113R mutation (*right*) and Scc2p-E822 due to close proximity. The *bottom* panels illustrate that Smc3p-K113 acetylation (*left*) or *K113Q* mutation (*right*) alters spacing, precluding a salt bridge. All structure illustrations in this report, including residue substitutions and alterations, were generated using PyMOL software. (*C*) *SCC2/SCC4* overexpression suppresses the inviability of the *smc3-K113Q* acetyl mimic. The haploid *SMC3-AID* strain also bearing either a wild-type *SMC3* (VG3919-3C), *smc3-K113Q* (KB62A), or *smc3-K113Q* containing *pGAL-SCC2/SCC4* (VG4052-3A) was grown at 30°C to saturation; plated at 10-fold serial dilutions on YPD, YPD + IAA, or YEPG + IAA; and incubated for 4 d at 23°C. (*D*) *SCC2/SCC4* overexpression fails to suppress the cohesion defect of *smc3-K113Q* cells. Strains in *C*, along with a haploid containing *SMC3-AID* as the sole *SMC3* (VG3651-3D), were arrested in G1, depleted for *SMC3-AID*, *SCC2/SCC4*-overexpressed, synchronously released from G1, and arrested in mid-M phase. Cells were fixed and processed to score cohesion at a chromosome IV arm locus using the LacO–LacI system as described in the Materials and Methods. The number of GFP spots was scored in mid-M-phase-arrested cells, and the percentage of cells with defective cohesion (two GFP spots) was plotted. Two-hundred cells were scored for each data point, and data were generated from two independent experiments. (*E*) Cohesin binding to chromosomes is greatly reduced in *smc3-K113Q* cells and only slightly increased by *SCC2/SCC4* overexpression. Aliquots of mid-M-phase cells from *D* were fixed and processed for ChIP using anti-Mcd1p antibodies as described in the Materials and Methods. Mcd1p binding was assessed by qPCR and is presented as a percentage of input DNA. (*Left* panel) The chromosome IV arm *CAR* region (*TRM1*). (*Middle* panel) The chromosome III pericentric region (*CARC1*). (*Right* panel) Regions immediately flanking *CEN14* and *CEN4*.

Cohesin is also regulated through the acetylation of its subunits by the Eco1p acetyltransferase. Eco1p acetylates two conserved lysines in the Smc3p head domain, which occurs after cohesin binds DNA in S phase (Supplemental Fig. S1; Rolef Ben-Shahar et al. 2008; Ünal et al. 2008; Zhang et al. 2008). In *S. cerevisiae,* these lysines are at positions 112 and 113. Substitution of arginine for K113 (K113R) or both K112 and K113 (K112R, K113R) prevents their acetylation, causing inviability and a failure to establish sister chromatid cohesion (Rolef Ben-Shahar et al. 2008; Ünal et al. 2008). *Eco1* mutants have the same defects as the s*mc3-K112R, K113R*, indicating that these two Smc3p lysines are key targets of Eco1p. Likewise, the viability of *smc3-K112R, K113R* and *eco1Δ* mutants can be restored by deleting the *WPL1* gene (Rolef Ben-Shahar et al. 2008; Rowland et al. 2009; Sutani et al. 2009). Wpl1p is a conserved cohesin inhibitor that removes cohesin from DNA (Kueng et al. 2006; Chan et al. 2012). Thus, one evolutionarily conserved function of K113 acetylation is to antagonize Wpl1p and stabilize cohesin's binding to DNA.

However, *WPL1* deletion minimally suppresses the sister chromatid cohesion defects of either *smc3-K112R, K113R* or *eco1Δ* mutants (Rowland et al. 2009; Sutani et al. 2009; Guacci and Koshland 2012; Guacci et al. 2015). Other mutations have been identified that do suppress the cohesion defects of acetyl-defective mutants (Çamdere et al. 2015; Guacci et al. 2015; Elbatsh et al. 2016). These mutations map to the Smc3 ATPase active site and reduce cohesin ATPase activity (Çamdere et al. 2015; Elbatsh et al. 2016). This observation suggests that cohesin's unacetylated and acetylated states impose different cohesin ATPase levels. Initially, the higher ATPase levels of unacetylated cohesin induced by Scc2p promote efficient cohesin DNA binding. Once cohesin is bound to a sister chromatid, Smc3p acetylation lowers cohesin's ATPase activity. The lower ATPase activity stabilizes its DNA binding and promotes the capture of the other sister chromatid to generate cohesion (Çamdere et al. 2015, 2018; Elbatsh et al. 2016). The mutants lacking Smc3p acetylation cannot stably bind DNA or capture a second DNA molecule because its ATPase activity is too high. This defect of *smc3-K112R, K113R* or *eco1Δ* mutants is counteracted by the suppressor mutations in the Smc3 ATPase active site that slow ATP hydrolysis (Çamdere et al. 2015, 2018).

How acetylation alters cohesin's ATPase has remained a mystery, as the Smc3p-K113 residue is not proximal to either of cohesin's ATPase active sites (Supplemental Fig. S1A). A recent study suggested that Smc3p-K113 acetylation inhibited Scc2p binding to cohesin indirectly by stabilizing the binding of Pds5p, which then precluded Scc2p binding (Bastié et al. 2022; van Ruiten et al. 2022). However, a more direct mechanism was suggested by the observation that Scc2p stimulation of cohesin ATPase activity in vitro is drastically reduced by a mutation that mimics acetylation by substituting glutamine for K113 (K113Q) (Murayama and Uhlmann 2015). Notably, this inhibition occurred in the absence of Pds5p, suggesting that acetylation controls cohesin ATPase by a second Pds5p-independent mechanism.

Here, we test and elaborate on this hypothesis by addressing three mechanistic questions: (1) Does acetylating Smc3p-K113 impede Scc2p function in vivo, as suggested by the in vitro experiments? (2) How does Scc2p activate cohesin's ATPase? (3) How is cohesin's ATPase activity repressed by acetylation of the Smc3-K113 residue, given that this residue lies far away from either of cohesin's ATPase active sites? The answers to these questions led us to a model that explains both Scc2p's activation of cohesin's ATPase and its inhibition by acetylation.

## Results

### Inhibiting the salt bridge between Smc3-K113 and Scc2p compromises cohesin function in vivo

Further clues into how Smc3p-K113 acetylation could impact Scc2p function came from the cryo-EM structures of yeast and human cohesin with Scc2p or its human ortholog, NIPBL (Collier et al. 2020; Shi et al. 2020). The Smc3p-K113 residue and its homologous residue in humans, Smc3p-K106 (Zhang et al. 2008), are positioned to make a salt bridge with the carboxyl group of conserved Scc2p glutamate (E822 in Scc2p and E1899 in human NIPBL) (Fig. 1B; Supplemental Fig. S1B,C). This salt bridge suggested that the K113 residue directly contributed to Scc2p's stimulation of cohesin's ATPase by facilitating Scc2p binding to the cohesin head.

The involvement of the Smc3p-K113 residue in this salt bridge provided a simple mechanism for how its acetylation, the acetyl mimic (smc3-K113Q), and the acetyl defect (smc3-K113R) could alter Scc2p's stimulation of the cohesin ATPase. The positively charged guanidino nitrogens of the K113R substitution should still be able to form the salt bridge with the negatively charged carbonyl oxygen of the Scc2p-E822 residue. To test this assumption, we made the arginine substitution in the cryo-EM structure. Indeed, the guanidino nitrogens were close enough (<4 Å) to form an effective salt bridge and should allow the binding of Scc2p and its stimulation of the cohesin ATPase (Fig. 1B). However, since the arginine substitution cannot be acetylated, the salt bridge should persist constitutively, causing the *smc3-K113R* mutant to phenocopy *eco1* mutants (Rolef Ben-Shahar et al. 2008; Ünal et al. 2008; Guacci and Koshland 2012).

Conversely, acetylation of the Smc3p-K113 residue or the smc3p-K113Q substitution would eliminate the lysine's positive charge and block the formation of the salt bridge with Scc2p. The disruption of the salt bridge would alter Scc2p's binding to cohesin such that it could no longer stimulate the ATPase (Murayama and Uhlmann 2015; Shi et al. 2020). While K113 acetylation and smc3p-K113Q would block the salt bridge, they would not generate structural conflicts that would dramatically impact the cohesin head structure (Fig. 1B).

To better understand the impact of Smc3p-K113 acetylation, we performed a detailed characterization of the *smc3-K113Q* mutant. For this purpose, we tagged the endogenous *SMC3* gene with an auxin-inducible degron (*SMC3-AID*). Next, we introduced a second *SMC3* allele: wild-type *SMC3* or *smc3-K113Q*. The addition of auxin (IAA) to the growth media for these cells induced the degradation of the Smc3p-AID protein, generating cells either lacking Smc3p (*SMC3-AID*) or containing only wild-type Smc3p (*SMC3-AID SMC3*) or only smc3p-K113Q (*SMC3-AID smc3-K113Q*). Cells lacking Smc3p or containing only smc3p-K113Q were inviable (Fig. 1C), corroborating a previous study showing that *smc3-K113Q* disrupts an essential cohesin function (Heidinger-Pauli et al. 2010).

To further characterize the *smc3-K113Q* mutant, we analyzed cells from these three strains for sister chromatid cohesion at a centromere-proximal and arm locus by fluorescently marking each sister chromatid with the LacO–lacI-GFP system (Marshall et al. 1997; Guacci et al. 2019). We depleted Smc3p-AID from G1 through M phase and assessed sister chromatid cohesion in mid-M-phase-arrested cells. The precocious sister chromatid separation was detected by the presence of two GFP spots. The percentage of cells with two spots increased dramatically in cells expressing only smc3p-K113Q compared with Smc3p, increasing threefold to fourfold near the centromere and 10-fold at the chromosome arm locus (Fig. 1D; Supplemental Fig. S1D). These cohesion defects were not as severe as in cells containing no Smc3p (Fig. 1D; Supplemental Fig. S1D). Thus, the acetyl-mimic mutant was severely but not completely defective for sister chromatid cohesion, as seen in previous studies (Ünal et al. 2008; Guacci and Koshland 2012).

Cohesin is bound to chromosomes at the centromeres, pericentric regions, and specific sites on chromosome arms from S phase to mid-M. To assess how Smc3p-K113Q affects cohesin binding to DNA, we performed chromatin immunoprecipitation followed by quantitative PCR (ChIP-qPCR) on aliquots of synchronously arrested cells in mid-M phase. Cells containing only Smc3p-K113Q showed a sixfold reduction in cohesin binding to an arm site, a twofold decrease at a pericentromeric site, and a variable loss of binding at centromeres compared with cells containing Smc3p (Fig. 1E). However, the Smc3p-K113Q cohesin binding levels at all these sites were well above those observed in cells lacking Smc3p, indicating that DNA binding of cohesin was significantly but not entirely compromised by Smc3p-K113Q. Taken together, the multiple defects of cells containing only Smc3p-K113Q were consistent with the idea that blocking the salt bridge between Smc3p-K113 and Scc2p inhibits Scc2p function and presumably its ability to stimulate cohesin ATPase.

Finally, we examined whether Smc3p-K113Q compromised the integrity of the cohesin core complex by assessing the levels of the Mcd1p subunit of cohesin in mid-M-arrested cells. Mcd1p is degraded unless associated with both Smc1p and Smc3p (Çamdere et al. 2015; Guacci et al. 2015, 2019; Robison et al. 2018). Mcd1p levels were reduced fourfold in Smc3p-K113Q cells compared with Smc3p but remained at least twofold higher than those lacking Smc3p (Supplemental Fig. S1E). This result suggested that the acetyl-mimic mutant partially destabilized Mcd1p's binding to cohesin.

Having completed this detailed characterization of the *smc3-K113Q* mutant, we assessed whether any of its phenotypes were suppressed by Scc2p/Scc4p overexpression as predicted if the acetyl-mimic mutation partially compromised Scc2p function. The inviability of the strain expressing only Smc3p-K113Q was suppressed by the galactose-induced overexpression of Scc2p and Scc4p (Fig. 1C). In contrast, Scc2p/Scc4p overexpression did not suppress the inviability of cells that were depleted for either Eco1p or the cohesion maintenance factor Pds5p (Supplemental Fig. S1F). These results are consistent with the hypothesis that Smc3p-K113 acetylation limited the function of Scc2p but not other cohesin regulators.

However, Scc2p/Scc4p overexpression minimally suppressed the Smc3p-K113Q-induced defects in cohesion, chromosome binding, and Mcd1p levels (Fig. 1D,E; Supplemental Fig. S1D,E). The ability of budding yeast to survive with minimal cohesion was not unexpected. We previously showed that budding yeast had the peculiar property of attaching its microtubules to the newly replicated sister kinetochores during S phase when only part of the chromosome arms had been replicated (Guacci and Koshland 2012). The unreplicated arm sequences acted as a surrogate for sister chromatid cohesion, thereby providing the tension needed for bipolar attachment of the sister chromatids. The presence of this surrogate allowed yeast with very little cohesion to survive in the unperturbed environment of the laboratory.

The *smc3-K113Q* mutant overexpressing Scc2p/Scc4p was also sensitive to benomyl (a broader measure of cohesin's mitotic function) and camptothecin (a measure of cohesin's function in DNA damage repair) (Supplemental Fig. S1G). The presence of these defects showed that Scc2p/Scc4p overexpression only partially restored cohesin function in the *smc3-K113Q* mutant. The inefficiency of suppression could be explained by the inability of Scc2p/Scc4p overexpression to restore normal DNA binding levels to cohesin with Smc3p-K113Q.

**Figure 2.**
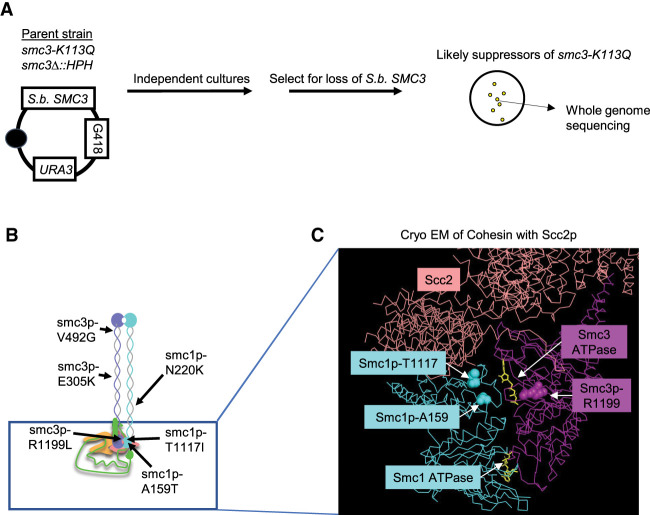
Suppressor screen linking the Scc2p–Smc3p-K113 interface with other Scc2p–cohesin head interfaces. Identification of suppressor mutations in *SMC1* and *SMC3* that suppress the lethality of *smc3-K113Q*. (*A*) Schematic of the genetic screen used to identify *smc3-K113Q* suppressors. The haploid strain (VG3969-14C) bears *smc3-K113Q* as the sole *S. cerevisiae* copy of *SMC3* and *S. bayanus SMC3* on a *CEN URA3 G418* plasmid (pFC3). Single colonies were grown to saturation in YPD and plated on FOA media to select for viable cells expressing only Smc3p-K113Q (see the Materials and Methods). Representative colonies from each plate were sequenced to identify the putative suppressor mutation. (*B*) Cartoon of the cohesin complex showing relative positions of the indicated *smc3-K113Q* suppressor mutations. (Cyan) Smc1p, (purple) Smc3p, (green) Mcd1p. Also depicted is cohesin-associating protein Scc2p (salmon). (*C*) Residues of three suppressors—Smc1p-R1199, Smc1p-T1117, and Smc1p-A159—map close to the Smc3p ATPase and Scc2p. Cryo-EM structure of the *S. cerevisiae* (PDB ID: 6ZZ6) (Collier et al. 2020) Smc1p (cyan) and Smc3p (magenta) head domains bound to Scc2p (salmon). The *S. cerevisiae* Smc3p-R1199 (magenta spheres), *S. cerevisiae* Smc1p-T1117 (cyan spheres), and *S. cerevisiae* Smc1p-A159 (cyan spheres) residues are indicated. ATP (yellow) and each SMC ATPase are also indicated.

To better understand how the *smc3-K113Q* mutation impacts Scc2p regulation of cohesin, we conducted a screen in budding yeast for suppressor mutations that restored viability to cells expressing only Smc3p-113Q (Fig. 2A). We hypothesized that these mutations would identify regions of cohesin that were responsive to Scc2p and/or Smc3p-K113 acetylation. We identified three suppressor mutations in *SMC3* and three in *SMC1* (Fig. 2B). All of the suppressor mutations were located in regions of the cohesin subunits that had conserved amino acid sequences (Supplemental Fig. S2A,B). We introduced each of the six suppressor mutations into the parental *smc3-K113Q* cells and also strains in an otherwise wild-type background (Materials and Methods). All six newly constructed double mutants were viable, demonstrating that our six mutations were responsible for the suppression of *smc3-K113Q* inviability. Thus, these mutations identified regions of the cohesin complex, which were functionally connected to an acetylated state of the Smc3p-K113 residue.

We mapped our suppressor mutations onto the cryo-EM structure of cohesin with the Scc2p complex (Collier et al. 2020). The six suppressor residues lay in distinct regions of cohesin: three in the Smc coiled-coil domains and three in the Smc head domains (Fig. 2B). This result suggested that multiple regions of cohesin were impacted by Smc3p-K113 acetylation (Fig. 2B). The three suppressors in the head were much closer to the Smc3p ATPase active site than the Smc1p ATPase active site (Fig. 2C). These results suggested that the acetylated state of Smc3p-K113 regulated cohesin by modulating the Smc3-ATPase active site.

Additional insights came from analyzing the local structure of the two residues that were mutated by the suppressors. The Smc1p-T1117 residue was proximal to an interface between Smc1p and Scc2p, and this interface and positioning were conserved between yeast and humans (Supplemental Fig. S2C). The Scc2p residues proximal to the Smc3p-R1199 residue were missing from the yeast structure; however, this Smc3p residue lay in a conserved region of the human Smc3p (Supplemental Fig. S2B). In the human structure, the conserved region of Smc3p was proximal to an interface between NIPBL (the Scc2p ortholog) and Smc3p (Supplemental Fig. S2D). The Smc residues of both of these interfaces were highly conserved, while the residues in NIPBL and Scc2p at these interfaces were not (Supplemental Fig. S2E–G). These results suggested that the Smc1–Scc2p and Smc3p–Scc2p interfaces were coevolving. Taken together, the positions of the suppressor residues suggested that alterations in these Smc1p–Scc2p or Smc3p–Scc2p interfaces could act at a distance to compensate for the defects associated with the elimination of the Scc2p–Smc3p-K113 salt bridge by K113 acetylation. This compensation suggested that proper Scc2p function involved functional cross-talk between three distinct interfaces of Scc2p and the cohesin head.

**Figure 3.**
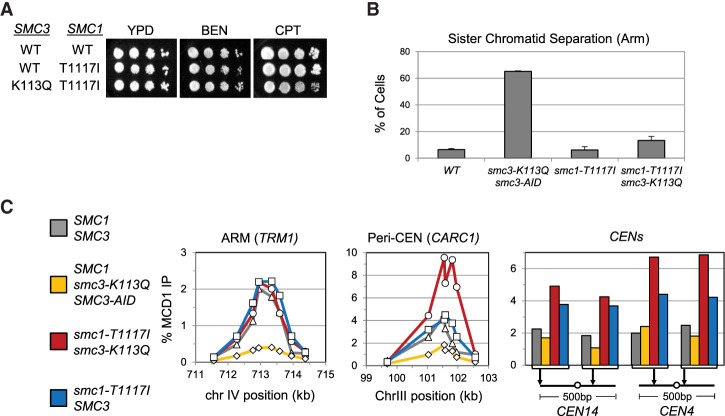
*Smc1-T1117I* uniquely and robustly suppresses *smc3-K113Q* defects in viability, drug sensitivity, cohesion, and cohesin binding to DNA. The *smc1-T1117I* mutant is a robust suppressor of the *smc3-K113Q* mutant. (*A*) The *smc1-T1117I smc3-K113Q* double mutant grows as well as WT and is resistant to drugs. The haploid wild-type (VG4012-2C), *smc1-T1117I* (VG4006-13A), and *smc3-K113Q smc1-T1117I* (VG4010-8B) strains were grown and diluted as described in Figure 1C; plated on YPD alone or containing 10 μg/mL benomyl (BEN) or 15 μg/mL camptothecin (CPT); and incubated for 3 d at 23°C, 4 d at 23°C, or 3 d at 30°C, respectively. Plates were electronically rearranged for ease of display. (*B*) *smc1-T1117I* strongly suppresses the cohesion defect of the *smc3-K113Q* mutant. The haploid wild-type (VG3620-4C), *smc3-K113Q smc3-AID* double-mutant (VG3891-6B), *smc1-T1117I* (VG4006-13A), and *smc3-K113Q smc1-T1117I* double-mutant (VG4010-8B) cells were arrested in G1; auxin was added to deplete Smc3p-AID; and cells were synchronously released from G1 and arrested in mid-M phase. Cohesion loss at a chromosome IV arm locus was assessed and plotted as described in Figure 1D. (*C*) The *smc1-T1117I smc3-K113Q* double-mutant cohesin binds to chromosomes at levels equal to or higher than wild-type cohesin. Mid-M-phase cells from *B* were fixed and processed for ChIP, and the level of cohesin bound to chromosomes was determined as described in Figure 1E. (*Left* panel) Chromosome IV arm *CAR* region (*TRM1*). (*Middle* panel) Chromosome III pericentric region (*CARC1*). (*Right* panel) Regions immediately flanking *CEN14* and *CEN4*.

To more fully evaluate the efficacy of these suppressors, we subjected strains with the suppressor mutation alone or with *smc3-K113Q* to the same battery of tests that we used to analyze suppression by Scc2p overexpression (Fig. 1C–E; Supplemental Fig. S1E,G). Five of the six double mutants behaved similarly: They grew slowly, exhibited significant benomyl and camptothecin sensitivity, and only partially restored cohesion (Fig. 3A,B; Supplemental Fig. S3A–D). The persistence of the phenotypes of these five mutants could have been caused by inefficient suppression of the *smc3-K113Q* allele or by new defects caused by the suppressor mutations. However, the suppressor mutations in an otherwise wild-type background had no obvious phenotypes (Supplemental Fig. S3A–D). Thus, the defects of the double mutants reflected the failure of these five suppressor mutations to fully suppress the defects in cohesin function caused by the *smc3-K113Q* mutation.

In stark contrast, the viability, growth, drug resistance, and cohesion of the *smc1-T1117I smc3-K113Q* double-mutant strain were nearly indistinguishable from the wild type (Fig. 3A,B). Furthermore, the cohesin binding to DNA in this double mutant was completely restored to wild-type levels at an arm locus and elevated above wild-type levels at the pericentromeric and centromere regions (Fig. 3C). Two of these mutants exhibited an increase in DNA binding at the centromere, possibly because these mutations affected the stability of cohesin binding to DNA (Eng et al. 2014). Thus, the *smc1-T1117I* suppressor compensated for almost all the biological and molecular defects imposed by *smc3-K113Q*. The only defect not completely restored in the *smc3-K113Q smc1-T1117I* double mutant was that Mcd1p levels were still reduced compared with wild type (Supplemental Fig. S3E,F). This result suggested that *smc1-T1117I* did not suppress *smc3-K113Q* mutant defects by simply increasing the level of mutant cohesin in cells. Rather, the *smc1-T1117I* mutation must have improved the functionality of Smc3p-K113Q cohesin.

### A model for Scc2p activation of cohesin's ATPase and its regulation by Smc3p-K113 acetylation

**Figure 4.**
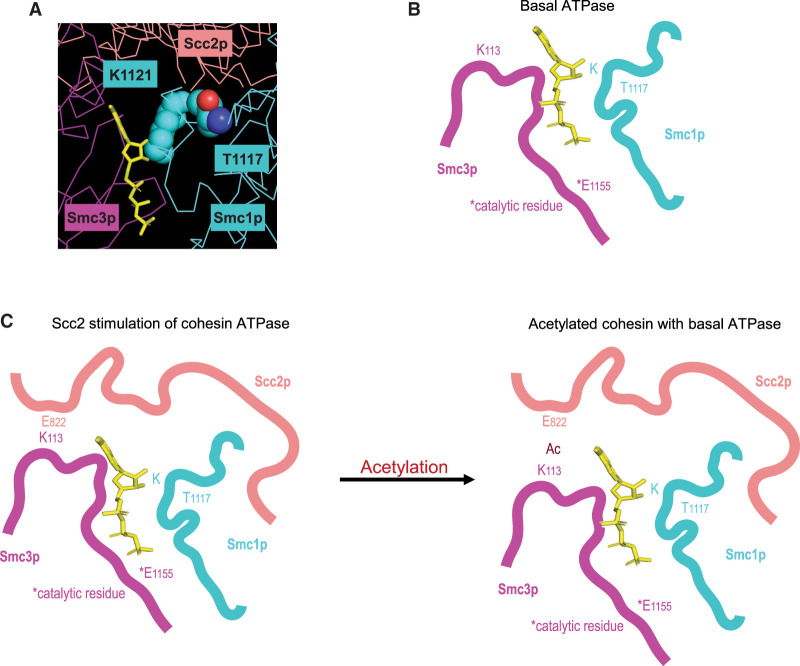
Model for Scc2p-mediated stimulation of Smc3p ATPase and its inhibition by acetylation. (*A*) Cryo-EM structure of the *S. cerevisiae* cohesin (PDB ID: 6ZZ6) (Collier et al. 2020) interface between Smc1p (cyan) and Scc2p (salmon), indicating Smc1p-T1117, Smc1p-K1121, and ATP (yellow). (*B*) Cartoon of the cohesin head domain in its basal ATPase state, with key Smc1p and Smc3p residues indicated. (*C*) Cartoon of the cohesin head domain in its stimulated ATPase state with Scc2p bound when Smc3p in not acetylated (*left*) and the inhibited state with Smc3p-K113 acetylated (*right*). (*Left*) Scc2p stimulation of cohesin ATPase: (1) Scc2p-E822 is in close proximity to unacetylated Smc3p-K113, which (2) properly orients the Scc2p–Smc1p interface. Scc2p binding at this Smc1p interface (3) shifts Smc1p and ATP such that (4) ATP is nearer the Smc3p-E1155 catalytic glutamate. (*Right*) Acetylated cohesin inhibits Scc2p stimulation of cohesin ATPase: (1) Acetylation of Smc3p-K113 disrupts Scc2p-E822 positioning, leading to (2) improper binding of Scc2p at the Smc1p-T1117 interface, resulting in (3) a failure to reposition the ATP closer to the catalytic glutamate, thereby (4) inhibiting Scc2p's stimulation of cohesin's ATPase.

We reasoned that *smc1-T1117I* improved *smc3-K113Q* cohesin function by overcoming the acetylation-dependent inhibition of Scc2p stimulation of cohesin's ATPase. A closer look at the cohesin–Scc2p cryo-EM structure provided an important clue as to how the suppressor mutation could impact ATPase activity. The Smc1p-T1117 residue was not only proximal to Scc2p but was also immediately adjacent to the Smc1p-K1121 residue, which was positioned to form a hydrogen bond with a hydroxyl group on the ribose ring of ATP in the Smc3 ATPase active site (Fig. 4A). Thus, the smc1p-T1117I suppressor identified a region of Smc1p that was positioned to both respond to Scc2p binding and impact an Smc1p residue (K1121) important for ATP binding.

These structural features of T1117, coupled with the functional features of the isoleucine suppressor substitution, led us to hypothesize the following model for Scc2p's activation of cohesin's ATPase and its regulation by Smc3p-K113 acetylation. Without Scc2p binding to cohesin, the binding of ATP in the Smc3p ATPase active site was not optimal for ATP hydrolysis (Fig. 4B). The binding of Scc2p at Smc3p-K113 helped orient Scc2p's binding to Smc1p. The binding of Scc2p at this Smc1p interface repositioned the neighboring Smc1p-K1121 and ATP such that ATP was more optimally positioned for hydrolysis by the catalytic glutamate (Smc3p-E1155) in the Smc3 ATPase active site (Fig. 4C, left). Acetylation (or the acetyl-mimic substitution) of Smc3p-K113 altered Scc2p binding at this interface and, consequently, Scc2p's binding at the Smc1p–Scc2p interface (Fig. 4C, right). The altered Scc2p binding to Smc1p failed to induce the repositioning of Smc1p residues for optimal ATP binding and hydrolysis. The isoleucine substitution for T1117 acted as a surrogate for proper Scc2p binding, repositioning the Smc1-K1121 residue and the associated ATP to improve ATPase activity. Our model for Scc2p's stimulation of cohesin's ATPase and its regulation by Smc3p-K113 acetylation led us to four predictions about the in vivo and in vitro consequences of Smc1p-T1117 substitutions.

### Only smc1p-T1117I and smc1p-T1117V mutants suppress the inviability of the acetyl mimic

**Figure 5.**
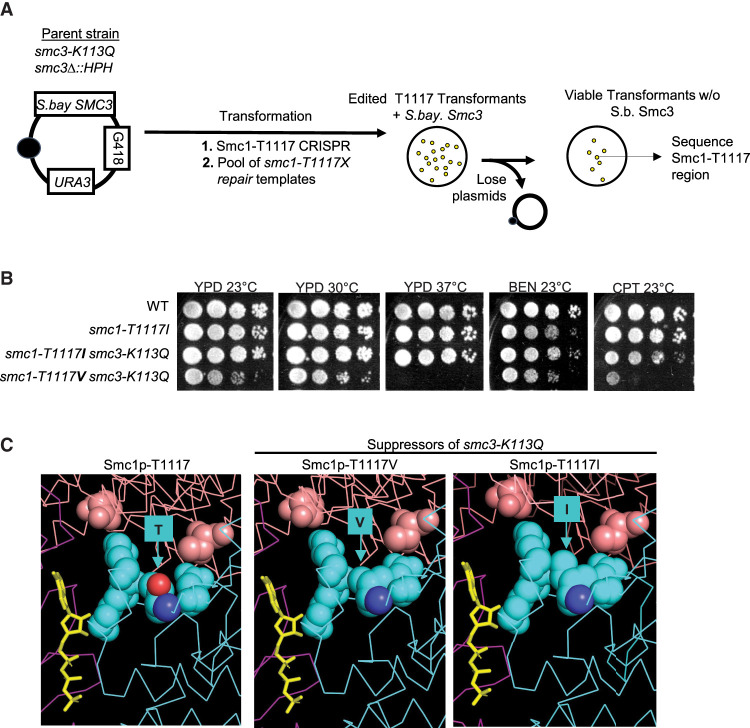
Only isoleucine or valine residue substitutions at *smc1-T1117* suppress *smc3-K113Q*. (*A*) Schematic of a screen to assess which substitutions at *smc1-T1117* suppress *smc3-K113Q*. The haploid strain (VG3969-14C) contains *smc3-K113Q* as the sole *S. cerevisiae SMC3* and *S. Bayanus SMC3* on a *CEN URA3 G418* plasmid (pFC3). CRISPR was used to insert random substitutions of the *smc1-T1117* residues as described in the Materials and Methods. Transformants were replica-plated to FOA media to select for loss of pFC3 (FOA^R^ G418^S^) colonies, which were sequenced to identify *smc1-T1117* substitutions that suppress *smc3-K113Q* inviability. (*B*) The valine substitution at *smc1-T1117* (T1117V) is a weaker suppressor of *smc3-K113Q* than isoleucine (T1117I). Wild-type (VG3620-4C), *smc1-T1117I* (VG4006-13A), *smc3-K113Q smc1-T1117I* (VG4010-8B), and *smc3-K113Q smc1-T1117V* (VG4147-14C) were grown and diluted as described in Figure 1C and plated on YPD and incubated for 4 d at 23°C or for 3 d at 30°C and 37°C or plated on YPD containing 10 μg/mL benomyl (BEN) or 15 μg/mL camptothecin (CPT) and incubated for 4 d at 23°C. (*C*) Model generated from the cryo-EM structure of *S. cerevisiae* cohesin (PDB ID: 6ZZ6) (Collier et al. 2020) depicting the interface between Smc1p-T1117 (cyan) and Scc2p (salmon). (*Left*) The interface between Scc2p and wild-type Smc1p. Structure models of Smc3p-K113Q suppressors Smc1p-T1117V (*middle*) and Smc1p-T1117I (*right*) are also shown.

Our model predicted that only a few substitutions of the Smc1p-T1117 residue would promote the necessary subtle alteration of its neighboring residues to increase ATP hydrolysis and suppress the acetyl-mimic mutant defects. Most substitutions at T1117 would fail to suppress the defects of the *smc3-K113Q* mutant because they either failed to change the K1121 position, leading to no change in the low ATPase activity, or radically changed the K1121 position, leading to even worse ATPase activity. To test this prediction, we made a library of DNA repair templates that encoded all possible 19 amino acid substitutions at T1117 (Fig. 5A). We assayed the ability of each of these substitutions to support the viability of otherwise wild-type cells (Supplemental Fig. S4) and to suppress the inviability of the *smc3-K113Q* mutant (Fig. 5B).

In the wild-type background, all substitutions except proline were viable and grew well at 23°C and 37°C (Supplemental Fig. S4). However, tyrosine, glutamate, and aspartate substitutions were sensitive to benomyl, and tyrosine substitution was sensitive to camptothecin, indicating that they partially compromised cohesin function. The remaining 15 substitutions were indistinguishable from the wild type for both drugs. By these criteria, most substitutions at *smc1-T1117* appeared to be compatible with generating enough ATPase activity to support most, if not all, of cohesin's in vivo functions. However, valine was the only substitution at T1117—besides isoleucine—that could suppress *smc3-K113Q* inviability, albeit more weakly than T1117I (Fig. 5B). The fact that only two structurally similar substitutions—isoleucine and valine—could suppress the acetyl mimic was consistent with a subtle structural change that would be needed to improve ATPase activity. Modeling of the valine and isoleucine substitutions in the cryo-EM structure revealed that these substitutions subtly filled in the space between the ATP binding K1121 and the residues contacting Scc2p (Fig. 5C). Thus, they could make a subtle change in the K1121 position that could alter ATP binding to improve its hydrolysis. In summary, these results showed that the *smc3-K113Q* suppressors at T1117 likely imposed a rare gain of function, consistent with the severe functional constraint of having to improve ATPase activity.

### The smc1p-T1117I substitution exacerbates the growth defect of acetyl-defective mutants

**Figure 6.**
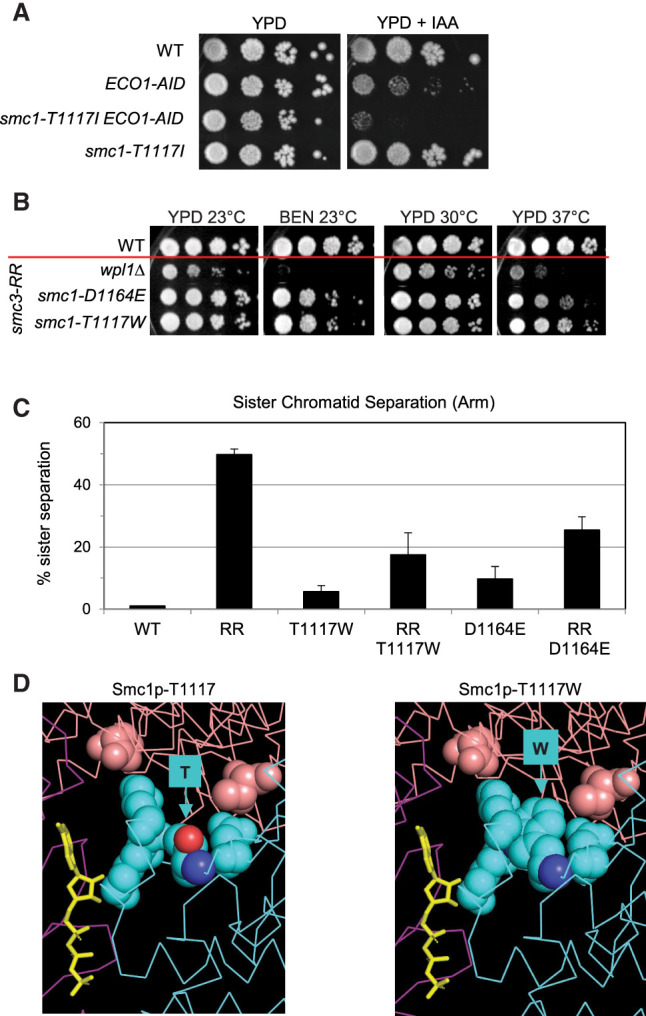
A tryptophan substitution at *smc1-T1117* (T1117W) strongly suppresses the *smc3-K112R, K113R* (acetyl-null) mutant. (*A*) *smc1-T1117I* exacerbates the growth defect of *ECO1-AID* depletion. Wild-type (VG3620-4C), *ECO1-AID* (VG3633-3D), *smc1-T1117I ECO1-AID* (KB118E), and *smc1-T1117I* (JL11A) strains were grown and diluted as in Figure 1C, plated on YPD and YPD + IAA, and incubated for 5 d at 23°C. (*B*) *smc1-T1117W* and *smc1-D1164E* strongly suppress *smc3-K112R, K113R* (RR). The haploid wild-type (VG3620-4C); *wpl1*Δ *smc3-K112R, K113R* (VG4154-3A); *smc1-D1164E smc3-K112R, K113R* (VG4153-5C); and *smc1-T1117W smc3-K112R, K113R* (VG4158-9D) strains were grown and diluted as in Figure 1C and plated on YPD and incubated for 4 d at 23°C, for 3 d at 30°C, or for 3 d at 37°C or plated on YPD containing 10 μg/mL benomyl and incubated for 5 d at 23°C (BEN 23°C). (*C*) *smc1-T1117W* strongly suppresses cohesion defect of the *smc3-K112R, K113R* mutant. The haploid wild-type (VG3620-4C); *smc3-AID smc3-K112R, K113R* (VG3991-1A); *smc1-T1117W* (VG4168-7B); *smc1-T1117W smc3-K112R, K113R* (VG4158-9D); *smc1-D1164E* (VG4138-1A); and *smc1-D1164E smc3-K112R, K113R* (VG4153-5C) strains were grown as described in Figure 3B and processed to assess cohesion loss as described in Figure 1D. (*D*) Model generated from the cryo-EM structure of *S. cerevisiae* cohesin (PDB ID: 6ZZ6) (Collier et al. 2020) illustrating wild-type Smc1p-T1117 (*left*) and acetyl-defective suppressor Smc1p-T1117W substitution (*right*).

Previous studies suggested that the inviability and cohesion defects of mutants with unacetylated cohesin resulted from the failure to down-regulate Scc2p stimulation of cohesin's ATPase activity (Çamdere et al. 2015; Murayama and Uhlmann 2015; Elbatsh et al. 2016). If so, the growth defects of the acetyl-defective mutants should be made worse by smc1p-T1117I because it would further enhance the toxic ATPase activity of the unacetylated cohesin. To test this prediction, we introduced the *smc1-T1117I* mutation into strains containing one of two conditional alleles of *ECO1*: either the temperature-sensitive *eco1-(ctf7-203)* or the auxin-sensitive *ECO1-AID* (Fig. 6A; Supplemental Fig. S5A). We then tested these double-mutant strains for their growth under conditions where Eco1p function was reduced, leading to the underacetylation of Smc3p-K113. The *smc1-T1117I* mutant not only failed to suppress the growth defects of the *eco1* temperature-sensitive allele or the auxin-sensitive allele but exacerbated them further (Fig. 6A; Supplemental Fig. S5A). This exacerbation fit with our model that the suppressor mutation altered the T1117 region of Smc1p to increase ATPase activity. This putative increase suppressed defects caused by the acetyl-mimic mutant's inhibition of ATPase stimulation but was toxic to cells with hyperactive ATPase generated when Smc3p-K113 acetylation levels were reduced.

### Substitutions of Smc1p-T1117 can also down-regulate cohesin's ATPase, acting as a surrogate for Smc3p acetylation

We reasoned that if the T1117 region of Smc1p was critical for mediating Scc2p's stimulation of the ATPase, then a different subset of substitutions at T1117 might alter this critical region to decrease cohesin's ATPase activity. These substitutions should behave similarly to Smc1p-D1164E, which also reduced cohesin ATPase activity and suppressed the in vivo defects of acetyl-defective mutants (Çamdere et al. 2015; Elbatsh et al. 2016).

To test this prediction, we asked whether any T1117 substitution could restore the biological and molecular functions of cohesin containing smc3p-K112R, K113R (Supplemental Fig. S5B). This acetyl-defective mutant was known to be inviable and have a severe cohesion defect (Ünal et al. 2008; Guacci et al. 2015). We identified eight substitutions at T1117 that suppressed the inviability of *smc3-K112R, K113R* (Supplemental Fig. S5C). Six of the eight substitutions (Ala, Asp, Glu, Gly, Ser, and Lys) failed to grow at 37°C. They were extremely sensitive to benomyl (Supplemental Fig. S5B). The partial suppression of the growth defects of the acetyl-defective mutants by these substitutions was consistent with their reducing the ATPase activity of unacetylated cohesin sufficiently to support slow growth but not to restore all of cohesin's biological functions.

In contrast, mutations to tryptophan (Trp) *smc1-T1117W* and phenylalanine (Phe) *smc1-T1117F* in the *smc3-K112R, K113R* background were nearly wild type in their growth at high temperatures and also showed significant resistance to benomyl (Supplemental Fig. S5C). We compared the strongest acetyl-defective suppressor, *smc1-T1117W*, with previously described acetyl-defective suppressors *wpl1*Δ and *smc1-D1164E*. *smc1-T1117W* suppressed the growth defects, benomyl sensitivity, and cohesion defects of *smc3-K112R, K113R* much better than *wpl1*Δ and slightly better than *smc1-D1164E* (Fig. 6B,C; Çamdere et al. 2015). Modeling of these tryptophan and phenylalanine substitutions at T1117 revealed that they clashed with the position of K1121 (Fig. 6D), providing a mechanism for how they could alter the positioning of the ATP in the active site and reduce its hydrolysis.

Taken together, our in vivo suppressor analyses showed that different subsets of T1117 substitutions could either enhance cohesin function to counter its down-regulation by acetylation or reduce cohesin function to counter its constitutive up-regulation by lack of acetylation. These results were consistent with our model that the T1117 region of Smc1p was capable of toggling cohesin's ATPase levels in response to proximal Scc2p binding and the acetylation state of the Smc3p-K113 residue.

### smc1p-T1117I increases cohesin ATPase activity

**Figure 7.**
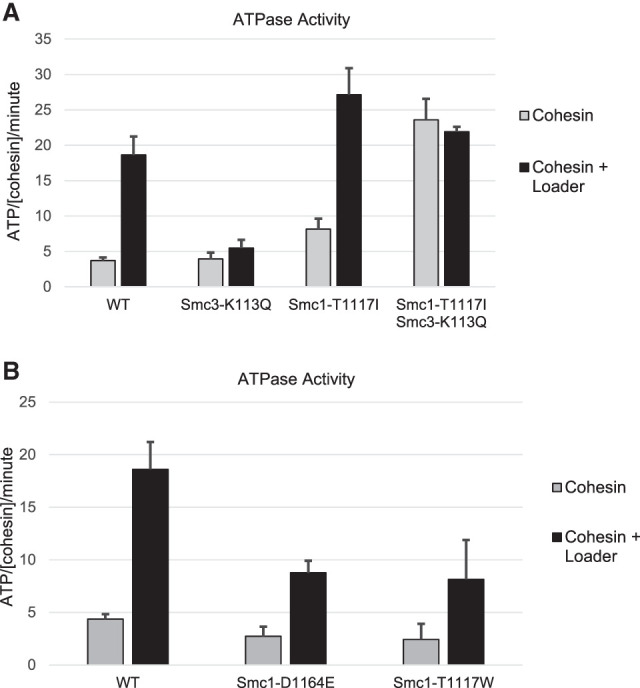
Different substitutions at *SMC1-T1117* up-regulate or down-regulate cohesin ATPase activity. (*A*) The Smc1p-T1117I suppressor of the Smc3p acetyl-mimic up-regulates cohesin ATPase activity. Wild-type and mutant cohesin complexes were purified and assessed for ATPase activity in the presence of DNA with and without Scc2p/Scc4p (loader). Purified cohesin concentration normalization was confirmed (Supplemental Fig. S6A). (*B*) The Smc1p-T1117W and Smc1p-D1164E suppressors of the Smc3 acetyl-null down-regulate cohesin ATPase activity. Wild-type and mutant cohesin complexes were purified and assessed for ATPase activity in the presence of DNA with and without Scc2p/Scc4p (loader). Purified cohesin concentration normalization was confirmed (Supplemental Fig. S6B).

Our model also predicted that cohesin's ATPase activity in vitro should be enhanced by Smc1p-T1117I. To test this prediction, we purified wild-type and mutant cohesins from an *eco1*Δ *wpl1*Δ strain that was alive but unable to acetylate cohesin. We measured their ATPase activity in the presence of DNA without Scc2p (basal) and with Scc2p (induced). We observed a similar basal ATPase activity for cohesin with wild-type Smc3p or Smc3p-K113Q (Fig. 7A,B). Scc2p addition stimulated ATPase activity of WT cohesin fourfold to fivefold but failed to stimulate Smc3p-K113Q cohesin as reported previously (Fig. 7A; Murayama and Uhlmann 2015). The presence of Smc1p-T1117I increased cohesin's basal ATPase activity about twofold (Fig. 7A). Addition of Scc2p increased ATPase activity of smc1p-T1117I cohesin to a level 50% greater than wild-type cohesin (Fig. 7A). For cohesin containing both Smc3p-K113Q and Smc1p-T1117I, the basal ATPase activity was dramatically increased relative to cohesin with just Smc3p-K113Q (Fig. 7A). This double-mutant cohesin reached ATPase levels greater than that seen for wild-type cohesin with Scc2p (Fig. 7A). Thus, as predicted from our model, the *smc1-T1117I* substitution restored cohesin ATPase activity to cohesin with the acetyl-mimic mutation.

As a corollary, our model also predicted that cohesin's ATPase should be inhibited by Smc1p-T1117W similar to Smc1p-D1164E. Cohesin with these two Smc1p mutants had similar levels of basal cohesin ATPase. Scc2p addition induced ATPase activity of both mutant complexes twofold less than wild type, similar to the levels previously reported for cohesin with Smc1p-D1164E (Elbatsh et al. 2016). Therefore, Smc1p-T1117W reduced cohesin ATPase activity, as our in vivo results and model predicted.

An unexpected observation from these studies was that the Scc2p-independent ATPase activity of cohesin with both Smc3p-K113Q and Smc1p-T1117I was significantly greater than cohesin with Smc1p-T1117I alone or Smc3p-K113Q alone. Thus, this very high level of basal ATPase activity required Smc3p-K113Q as well as the Smc1p-T1117I substitution. This result suggested that the acetylation state of Smc3p-K113 could potentially have additional impact on cohesin's ATPase activity beyond modulating Scc2p function.

The increase in Scc2p-independent ATPase activity of cohesin by Smc1p-T1117I also suggested that this substitution might reduce, or perhaps even bypass, the need for Scc2p in vivo. To test this possibility, we introduced the *smc1-T1117I* allele into cells harboring either the auxin-sensitive *SCC2-AID* or temperature-sensitive *scc2-4* alleles. The growth on auxin (IAA)-containing media of the *SCC2-AID smc3-T1117I* mutant was significantly greater than the *SCC2-AID* mutant but not restored to wild-type growth (Supplemental Fig. S6C). The temperature-sensitive growth of *scc2-4* was also partially suppressed by *smc1-T1117I*, as evidenced by growth at 30°C but not 35°C (Supplemental Fig. S6D). However, the *SCC2-AID smc1-T1117I* double mutant was nearly identical to *SCC2-AID* alone, as both strains exhibited extremely poor cohesion and very low cohesin binding to chromosomes (Supplemental Fig. S6E–G). Finally, the *smc1-T1117I smc3-K113Q* double mutant was unable to suppress a loss of Scc2p (Supplemental Fig. S6H). Together, these results suggested that higher basal ATPase of *smc1-T1117I* cohesin partially compensated for reduced Scc2p function, but even the hyperactive s*mc3-K113Q smc1-T1117I* cohesin cannot bypass the need for Scc2p.

## Discussion

In this study, we sought to answer three mechanistic questions about cohesin regulation. How does Scc2p activate cohesin's ATPase? Does acetylating Smc3p-K113 impede the ability of Scc2p to activate cohesin ATPase in vivo, as suggested by in vitro experiments? How is cohesin's ATPase activity repressed by Smc3p-K113 acetylation, given that this residue is not proximal to either of cohesin's ATPase active sites? To answer these questions, we isolated suppressors of the inviability of *smc3-K113* acetyl-mimic or acetyl-null mutants. We assayed the impact of these mutant proteins on cohesin function through in vitro ATPase assays, as well as in vivo assays of cohesion, chromosome-binding, drug resistance, and cohesin structural integrity. We also interrogated how suppressors impacted cohesin structure using the cryo-EM structure of cohesin with Scc2p. From our results, we developed a model for Scc2p stimulation of cohesin ATPase and its regulation by acetylation of the Smc3-K113 residue. Below, we summarize the model and our key observations that support the model.

We propose that formation of the Scc2p–Smc1p interface causes a shift in the neighboring Smc1p residues and the nearby ATP in the Smc3p ATPase active site. This shift better orients the ATP for hydrolysis by the catalytic glutamate of Smc3p-E1155. Activation of the Smc3p ATPase by the Scc2p–Smc1p interface is highly dependent on the positioning of Scc2p binding to the cohesin head. This positioning is determined by the Scc2p–Smc3p interfaces around Smc3p residues K113 and likely R1199. Acetylation of Smc3p-K113 directly alters Scc2p's interface with Smc3p, which in turn alters Scc2p's interface with Smc1p residues proximal to the Smc3p ATPase site. These acetylation-mediated alterations inhibit Scc2p's ability to shift the key residues in Smc1p that promote ATP hydrolysis.

This model is based on four key observations. First, we showed that overexpression of the Scc2p–Scc4p complex weakly suppressed the inviability of the *smc3-K113Q* mutant (acetyl mimic). This result indicates that the acetylation of cohesin limits Scc2p function in vivo, as had been suggested by a cryo-EM structure and in vitro studies of Scc2p activation of cohesin ATPase (Murayama and Uhlmann 2014; Shi et al. 2020). This partial suppression by Scc2p overexpression also suggests that Smc3p-K113 acetylation does not abolish Scc2p binding to the cohesin head but rather alters the interaction. Second, suppressors of the acetyl mimic that lay in the Smc head domains were proximal to the Smc3p ATPase active site, suggesting that Smc3p acetylation regulated cohesin specifically by modulating the Smc3p ATPase activity. Similarly, previously identified suppressors of Smc3p acetyl-defective mutants also lay proximal to the Smc3p ATPase (Çamdere et al. 2015; Elbatsh et al. 2016). Third, we demonstrated that substitutions of the Smc1p-T1117 residue were capable of toggling cohesin's ATPase levels up or down, suppressing Smc3p acetyl-mimic or acetyl-defective mutants, respectively. This residue is proximal to Smc1p residues that interface with Scc2p and help position ATP in the Smc3p ATPase active site. Structural modeling of these substitutions in the cryo-EM structure reveals that they would cause subtle changes that alter ATP positioning in the Smc3p ATPase active site, consistent with their impact on ATPase activity. Thus, it is reasonable to propose that Scc2p binding proximal at this Smc1p region could induce subtle structural changes similar to those induced by the acetyl-mimic suppressors to enhance ATPase activity. Finally, different substitutions at *smc1-T1117* suppress the inviability of the acetyl-mimic and acetyl-defective mutants of Smc3p-K113. This fact connects acetylation-dependent alterations in Scc2p's interaction with the Smc3p-K113 residue to the distal interactions of Scc2p with the critical Smc1p region that controls ATPase activity.

Our model raises a conundrum. Why not control the ATPase by modifying the Scc2p–Smc1p interface, which directly modulates ATPase activity, rather than by modifying a distal interface between Scc2p and the Smc3p-K113 residue? One possibility is that differentially regulating cohesin's loop extrusion, stable loop formation, and cohesion functions requires communication between biochemical activities in the Smc3p–Scc2p region and the Smc3p ATPase. Indeed, a recent study suggests a model for loop extrusion (Bauer et al. 2021) in which DNA is entrapped between Scc2p and the cohesin head near the Smc3p-K113–Scc2p and also binds at the hinge. Transfer of DNA from the hinge to the head is thought to be dependent on Scc2p's stimulation of cohesin's ATPase. Cycles of ATP binding and hydrolysis would allow the cycles of DNA release and recapture that are needed for loop extrusion. Inhibiting Scc2p's stimulation of cohesin's ATPase by Smc3p-K113 acetylation would trap DNA binding to the hinge, generate a stable DNA loop from loop extrusion, or generate cohesion if the hinge is bound to the sister chromatid. Thus, sensing DNA binding in the Smc3p-K113 region could be an important input to stimulate ATPase for loop extrusion and the acetylation-dependent entrapment of DNA needed for tethering.

Another conundrum arises from our suppressor analysis. In wild-type cells, only a fraction of cohesin is acetylated, presumably to generate two pools of cohesin: one with high ATPase for looping and another with low ATPase for cohesion. However, the *smc1-T1117I* and s*mc1-T1117W* mutations allow near-perfect suppression of the defects caused by acetyl-mimic and acetyl-null mutations, respectively. These results suggest that all cohesin's biological functions can be carried out by cohesin in a single acetylated state with ATPase activity either lower (*smc3-K112R, K113R smc1-T1117W* or *smc3-K113R smc1-D1164E*) or higher (*smc1-T1117I smc3 -K113Q*) than wild type. One possibility is that the ATPase and its biological functions are controlled by an additional regulatory protein like Pds5p (Bastié et al. 2022; van Ruiten et al. 2022). However, invoking redundancy avoids the question of what advantage in fitness acetylation-dependent control of cohesin's ATPase provides. A clue may come from the one residual phenotype in these cells with fixed ATPase levels: They exhibit some sensitivity to DNA-damaging agents. It will be exciting to use the different suppressors to probe the impact of the different acetylation and ATPase states on DNA repair and chromosome structure.

## Materials and methods

### Yeast strains, media, and reagents

Yeast strains used in this study were A364A background unless otherwise specified. Genotypes are listed in Supplemental Table S1. YPD media was made as previously described (Guacci et al. 1997). YEPR and YEPG were the same as YPD except they contained 2% raffinose or galactose instead of dextrose. YEPRG had 2% galactose and raffinose. Plates containing benomyl or camptothecin (Sigma C9911), used to assess drug sensitivity, were prepared as previously described (Guacci and Koshland 2012). Auxin (3-indoleacetic acid; Sigma-Aldrich) was made as a 1 M stock in DMSO and then added to 500 μM or 750 μM final concentration in liquid media or plates, respectively.

#### Cohesin purification media

Low biotin synthetic complete (LBSC) media contained 1.56 g/L BSM powder (Sunrise Science Products 1387), 1.71 g/L YNB-biotin powder (Sunrise Science Products 1523), 38 mM ammonium sulfate (5 g/L), 1 nM D-biotin (Invitrogen B20656), and 2% raffinose.

#### Cohesin loader purification media

Low biotin URA-dropout media contained 0.8 g/L CSM-Ura (Sunrise Sience Products), 1.71 g/L YNB-biotin powder (Sunrise Science Products 1523), 38 mM ammonium sulfate (5 g/L), 1 nM biotin, and 2% raffinose.

### Dilution plating spot assays

Cells were grown to saturation in YPD media at 23°C or 30°C, plated in 10-fold serial dilutions on YPD alone or containing drugs, and then incubated at 23°C or 30°C.

### G1 arrest and synchronous release into mid-M-phase arrest

#### G1 arrest

Asynchronous mid-log cultures were arrested in G1 by addition of α factor as previously described (Guacci et al. 2019). When required, auxin was added (500 μM final concentration) to G1-arrested cells and incubated for 30 min while arrested in G1. To induce *pGAL* promoters, galactose was added to 2% final concentration, and cells were incubated for 30 min.

#### Synchronous release from G1 into mid-M-phase arrest

G1-arrested cells were released from G1 into either YPD or YEPRG containing nocodazole and Pronase E as previously described (Guacci et al. 2019) and then incubated for 2.5 h at 30°C for YPD or 4 h at 30°C for YEPRG to arrest in mid-M phase. When required, auxin was added (500 μM final concentration) to all wash media and to resuspension media to ensure AID-tagged protein depletion.

### Protein extracts and Western blotting

#### Total protein extracts

Two to four OD_600_ cell equivalents were frozen and protein extracts were made as described in Guacci et al. (2019).

#### Western blots

Protein extracts were loaded onto 8% SDS-PAGE gels, subjected to electrophoresis, and then transferred to PDVF membranes. Proteins were detected using HRP-conjugated antibodies.

#### Chromatin immunoprecipitation (ChIP)

Aliquots of cells synchronously arrested in mid-M phase were fixed and processed for ChIP as described previously (Guacci et al. 2019). Primers used for ChIP are listed in Supplemental Table S3.

### Monitoring cohesion using LacO-GFP assay

Cohesion was monitored at *CEN*-proximal and *CEN*-distal loci using the LacO–LacI system as previously described (Guacci and Koshland 2012). Mid-M-phase cells were fixed, and the number of GFP signals in each cell was scored. Cells with two GFP spots have defective cohesion. G1-arrested cells were scored to confirm that mid-M-phase cells with two GFP spots were not due to pre-existing aneuploidy.

#### Microscopy

All images were collected on a Zeiss Axio Observer Z1 microscope equipped with a plan-apochromat 63× objective and the Definite focus system to maintain focus over time. *Z*-series optical sections were collected with a step size of 0.2 µm using an ASI MS-2000 XYZ piezo stage. Multiple stage positions were collected. The microscope, camera, and stage were controlled with the µManager software (Edelstein et al. 2014). *Z*-series were viewed using Fiji software (Schindelin et al. 2012).

#### Flow cytometry

Flow cytometry analysis was performed as previously described (Bloom et al. 2018), except that the fixed cells were washed twice in 1× TE with 0.2% (v/v) Tween-20.

#### CRISPR-mediated strain building

CRISPR guide plasmids and PCR-generated repair templates were made and used to insert mutations into yeast as previously described (Saxton and Rine 2019). CRISPR guides and repair templates are listed in Supplemental Tables S2 and S3.

### Isolation of spontaneous suppressors of the *smc3-K113Q* mutant

#### Screen for suppressors

Haploid VG3969-14C contained pFC3 (*S. bayanus SMC3 CEN URA3 G418*), endogenous *SMC3* deleted (*smc3*Δ*::HPH*), and *smc3-K113Q* integrated at *LEU2* (pVG419 K113Q). The *S. bayanus SMC3* gene supports viability and prevents gene conversion of *smc3-K113Q*. Single colonies were grown to saturation in YPD, washed with H_2_O, plated on 5-FOA (FOA), and incubated at 30°C. FOA^R^ G418^S^ colonies have lost pFC3. PCR sequencing confirmed that *smc3-K113Q* remained, so colonies had suppressor mutations. These were sequenced to identify the suppressor.

#### Rebuilding strains to confirm suppressors

##### *SMC3* suppressors

Haploid 3961-4B (*smc3*Δ*::HPH* + pFC3 [*S. bayanus SMC3 CEN URA3 G418*]) was transformed with PpuMI-linearized *LEU2* plasmids containing WT *SMC3* (pVG419), a suppressor allele alone, or suppressor with *smc3-K113Q*. pFC3 was lost by plating strains on 5-FOA media. Mutants were confirmed by PCR sequencing.

##### *SMC1* suppressors

Haploid 3965-1A (*smc1*Δ*::HPH* + pFC1 [*S. bayanus SMC1 CEN URA3 G418*]) was transformed with PpuMI-linearized *LEU2* plasmids containing WT *SMC1* (pVG444) or bearing the suppressor *smc1* alleles. CRISPR was used to insert *smc3-K113Q* at the endogenous locus in strains, and then pFC1 was lost by plating on 5-FOA media. Mutants were confirmed by PCR sequencing.

### Screen for *smc1-T1117* residues that suppress *smc3-K113Q*

CRISPR was used to insert random residues at *smc1-T1117* (PCR repair *smc1-T1117X*) in haploid 3961-4B (*smc3-K113Q-LEU2:leu2-3112 smc3*Δ*::HPH* + pFC3 [*S. bayanus SMC3 CEN URA3 G418*]). Transformants were plated on media to select for CRISPR plasmid, and colonies were replica-plated to FOA media. FOA^R^ colonies had lost pFC3 and thus contained *smc1-T1117* substitutions that suppress *smc3-K113Q.* PCR sequencing confirmed that *smc3-K113Q* remained and identified which *smc1-T1117* residue substitutions are suppressors. Thirteen strong (drug-resistant) and nine weak (drug-sensitive and temperature-sensitive) suppressors were sequenced. All strong suppressors were isoleucine (T1117I), and all weak suppressors were valine (T1117V).

### Assessing which substitutions at *smc1-T1117* support viability in WT cells

CRISPR was used to insert random residues at *smc1-T1117* (*smc1-T1117X*) into wild-type haploid 3620-4C. PCR sequencing of transformant colonies identified *smc1-T1117* residues that support viability.

### Screening for *smc1-T1117* residues that suppress *smc3-K112R, K113R*

CRISPR was used to insert random residues at *smc1-T1117* (*smc1-T1117X*) into haploid 4144-5C (*smc3-K112R,K113R-LEU2:leu2-3112, smc3*Δ*::HPH* + pFC3 [*S. bayanus SMC3 CEN URA3 G418*]). *Smc1-T1117* residues that suppress *smc3-K112R, K113R* were identified as described above to identify for *smc1-T1117* residues that suppress *K113Q*.

### Sequence alignment

Protein amino acid sequences were aligned in AliView using multiple sequence comparison by log expectation (MUSCLE).

### Protein purification

#### Cohesin purification

Cohesin purification strains were grown in low biotin synthetic complete media containing 2% raffinose to OD 1.0 at 30°C. Galactose was added to 2% to induce protein expression and incubated for 2 h at 30°C. Cells were collected by centrifugation, washed once with cold PBS, washed with lysis buffer (50 mM HEPES at pH 7.5, 10% [v/v] glycerol, 150 mM NaCl, 5 mM MgCl_2_, 0.1 mM CaCl_2_), pelleted, and then frozen. The frozen pellet was thawed on ice and resuspended in lysis buffer containing 1.2% Igepal CA-630, 20 mM β-mercaptoethanol, and EDTA-free protease inhibitor tablet (Sigma). DNase I (Millipore-Sigma 11284932001) was added to a final concentration of 0.05 mg/mL, and then PMSF was added to a final concentration of 0.25 mM. Cells were lysed by sonication, and then lysate was clarified by centrifugation at 20,000*g* for 45 min at 4°C. Clarified lysate was loaded onto a StrepTrap XT column (Cytiva) pre-equilibrated with lysis buffer. The column was washed with 10 column volumes of lysis buffer and then eluted with six column volumes of elution buffer 1 (50 mM HEPES at pH 7.5, 10% [v/v] glycerol, 2 mM MgCl_2_, 300 mM NaCl, 50 mM D-biotin, 20 mM β-mercaptoethanol). Eluate was loaded onto a HiTrap Heparin HP column (Cytiva) and then eluted using five column volumes of elution buffer 2 (50 mM HEPES at pH 7.5, 10% [v/v] glycerol, 2 mM MgCl_2_, 800 mM NaCl, 20 mM β-mercaptoethanol). Only the last four column volumes were collected. NaCl concentration was adjusted to 266 mM NaCl, and then protein was concentrated by ultrafiltration (Thermo Scientific Pierce Protein Concentrater PES 30K PI88529S).

#### Cohesin loader purification

The loader purification strain was grown in low biotin URA-dropout media containing 2% raffinose to OD 1.0 at 30°C. Galactose was added to 2% to induce protein expression and incubated for 2 h at 30°C. Cells were collected by centrifugation, washed once with cold PBS, and then washed with lysis buffer (50 mM HEPES at pH 7.5, 10% [v/v] glycerol, 150 mM NaCl, 5 mM MgCl_2_, 0.1 mM CaCl_2_), and the pellet was frozen. The frozen pellet was thawed on ice and resuspended in lysis buffer containing 1.2% Igepal CA-630, 20 mM β-mercaptoethanol, and EDTA-free protease inhibitor tablet (Sigma). DNase I was added to a final concentration of 0.05 mg/mL, and then PMSF was added to a final concentration of 0.25 mM. Cells were lysed by sonication, and then lysate was clarified by centrifugation at 20,000*g* for 45 min at 4°C. Clarified lysate was loaded onto a StrepTrap XT column pre-equilibrated with lysis buffer. The column was washed with 10 column volumes of lysis buffer followed by 15 column volumes of STW buffer 300 (50 mM HEPES at pH 7.5, 10% [v/v] glycerol, 2 mM MgCl_2_, 300 mM NaCl) and then eluted with five column volumes of LE buffer 1 (50 mM HEPES at pH 7.5, 20% glycerol, 5 mM MgCl_2_, 300 mM NaCl, 50 mM biotin, 20 mM β-mercaptoethanol). Eluate was loaded onto a HiTrap Heparin HP column (GE Healthcare), washed with six column volumes of LHW buffer 300 (50 mM HEPES at pH 7.5, 20% [v/v] glycerol, 2 mM MgCl_2_, 300 mM NaCl), and then eluted using five column volumes of LE buffer 2 (50 mM HEPES at pH 7.5, 20% [v/v] glycerol, 2 mM MgCl_2_, 800 mM NaCl, 20 mM β-mercaptoethanol). Only the last four column volumes were collected. NaCl concentration was adjusted to 266 mM NaCl using LHW buffer 0 (50 mM HEPES at pH 7.5, 20% [v/v] glycerol, 2 mM MgCl_2_, 20 mM β-mercaptoethanol), and protein was concentrated by ultrafiltration (Thermo Scientific Pierce Protein Concentrator PES 30K PI88529S).

#### ATPase assay

ATPase activity of cohesin was measured using EnzChek phosphate assay kit with purified recombinant proteins depleted of free phosphate using inorganic phosphate binding resin (Abcam ab270547). Reactions were assembled with 10 nM cohesin, 15 nM Scc3 alone or with 65 nM Scc2/4, 0.1 mg/mL BSA, and 450 nM 60-mer dsDNA in ATPase reaction buffer (25 mM HEPES at pH 7.5, 20% glycerol, 50 mM NaCl, 1 mM MgCl_2_); reactions were initiated with addition of ATP to a final concentration of 1 mM. Spectrophotometric measurements at 360 nM were taken every 1 min for 2 h at room temperature. ATPase activities were calculated by linear regression of the raw data using GraphPad Prism software.

## Supplementary Material

Supplemental Material
