# Supplementary material for: A model for Scc2p stimulation of cohesin's ATPase and its inhibition by acetylation of Smc3p
Source: Genes Dev. 2023 Apr 1;37(7-8):277–90. doi: 10.1101/gad.350278.122 (PMC10153460; doi:10.1101/gad.350278.122)
Supplement: Supplemental Material [file supp_37_7-8_277__DC1.html]

A model for Scc2p stimulation of cohesin's ATPase and its inhibition by acetylation of Smc3p — A model for Scc2p stimulation of cohesin's ATPase and its inhibition by acetylation of Smc3p — Supplemental Material 

# A model for Scc2p stimulation of cohesin's ATPase and its inhibition by acetylation of Smc3p

## Supplemental Material

- Supplemental\_Figures.pdf
- Supplemental\_Table\_1.pdf
- Supplemental\_Table2List.docx
- Supplemental\_Table3List.docx
