# Supplementary material for: A model for Scc2p stimulation of cohesin's ATPase and its inhibition by acetylation of Smc3p
Source: Genes Dev. 2023 Apr 1;37(7-8):277–90. doi: 10.1101/gad.350278.122 (PMC10153460; doi:10.1101/gad.350278.122)
Supplement: Supplemental Material [file supp_gad.350278.122_Supplemental_Figures.pdf]

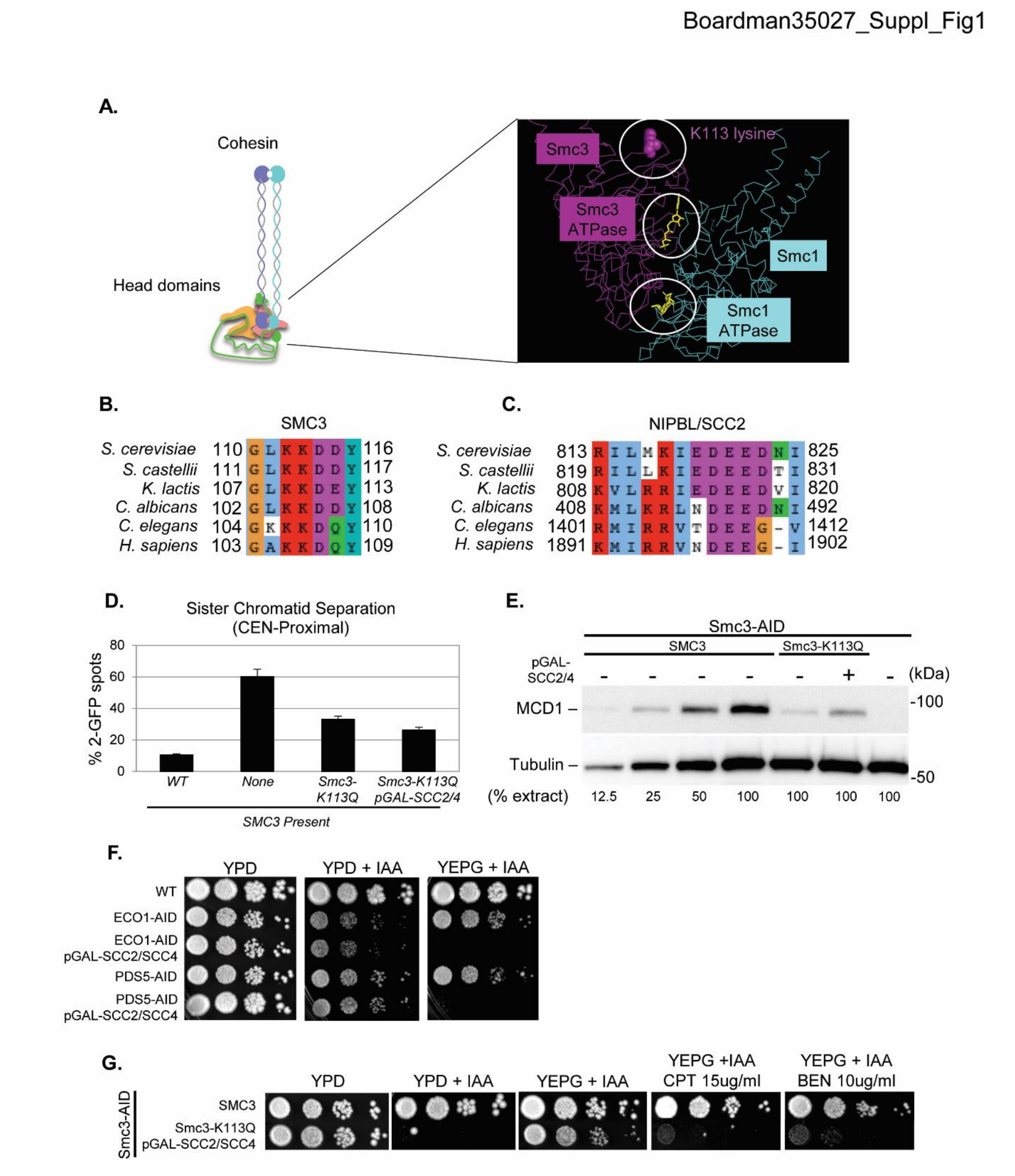

Figure 1S

(A) Cartoon depiction of cohesin (left). *S. cerevisiae* cohesin cryo-EM structure (PDB ID: 6ZZ6, Collier et al. 2020) of the head domains of Smc1p (cyan) and Smc3p (magenta) with ATPases, ATP (yellow) and the Smc3-K113 residue shown (right).

(B) Amino acid sequence alignment of the *S. cerevisiae* Smc3p-K113 region shown in Figure 1B with other Smc3p orthologs including *H. sapiens* Smc3p.

(C) Amino acid sequence alignment of the *S. cerevisiae* Scc2p-E822 region shown in Figure 1B with other Scc2p orthologs including *H. sapiens* NIPBL.

(D) *SCC2/SCC4* overexpression fails to suppress the *CEN*-proximal cohesion defect of *smc3-K113Q* cells. Haploid strains bearing *SMC3-AID* alone (JL16A) or also containing wild-type *SMC3* (JL17A), *smc3-K113Q* (JL14A), or *smc3-K113Q* with pGAL-*SCC2/SCC4* (JL15A) were grown and fixed as in Figure 1D. Strains contained LacO 10kb from *CEN4* to assess cohesion at a CEN-proximal locus. Cohesion was assessed as described in Figure 1D.

(E) Mcd1p levels are reduced when the *smc3-K113Q* acetyl-mimic mutant is the sole Smc3p present, but *SCC2/SCC4* overexpression partially restores Mcd1p levels. Protein extracts (TCA lysed) from 2 OD of mid-M phase cells in Figure 1D and 1E were analyzed by Western blot. Mcd1p protein levels were monitored using rabbit antibodies against Mcd1p (αMCD1) and rabbit antibodies against tubulin (αTUB2) as a loading control.

(F) *SCC2/SCC4* overexpression does not suppress *ECO1-AID or PDS5-AID* growth defects. Haploid wild-type containing pGAL-*SCC2/SCC4* (KB40A), *ECO1-AID* alone (VG3633-2D), *ECO1-AID* containing pGAL-*SCC2/SCC4* (KB50A), *PDS5-AID* alone (VG3862-1A), and *PDS5-AID* containing pGAL-*SCC2/SCC4* (KB46A), were grown were grown and dilution plated as in Figure 1C on YPD, YPD + IAA, or YEPG + IAA and incubated for 3 days at 23°C. Plates were electronically rearranged for ease of display.

(G) *SCC2/SCC4* overexpression partially suppresses the inviability of *smc3-K113Q*. Haploid *SMC3-AID* strain also bearing either a wild-type *SMC3* (VG3919-3C), or *smc3-K113Q* containing *pGAL-SCC2/SCC4* (VG4052-3A), were grown and diluted as in Figure 1C then plated on YPD, YPD + IAA, or YEPG+ IAA, YEPG + IAA + camptothecin [CPT] (15mg/ml), YEPG + IAA + benomyl [BEN] (10mg/ml) and incubated for 4 days at 23°C.

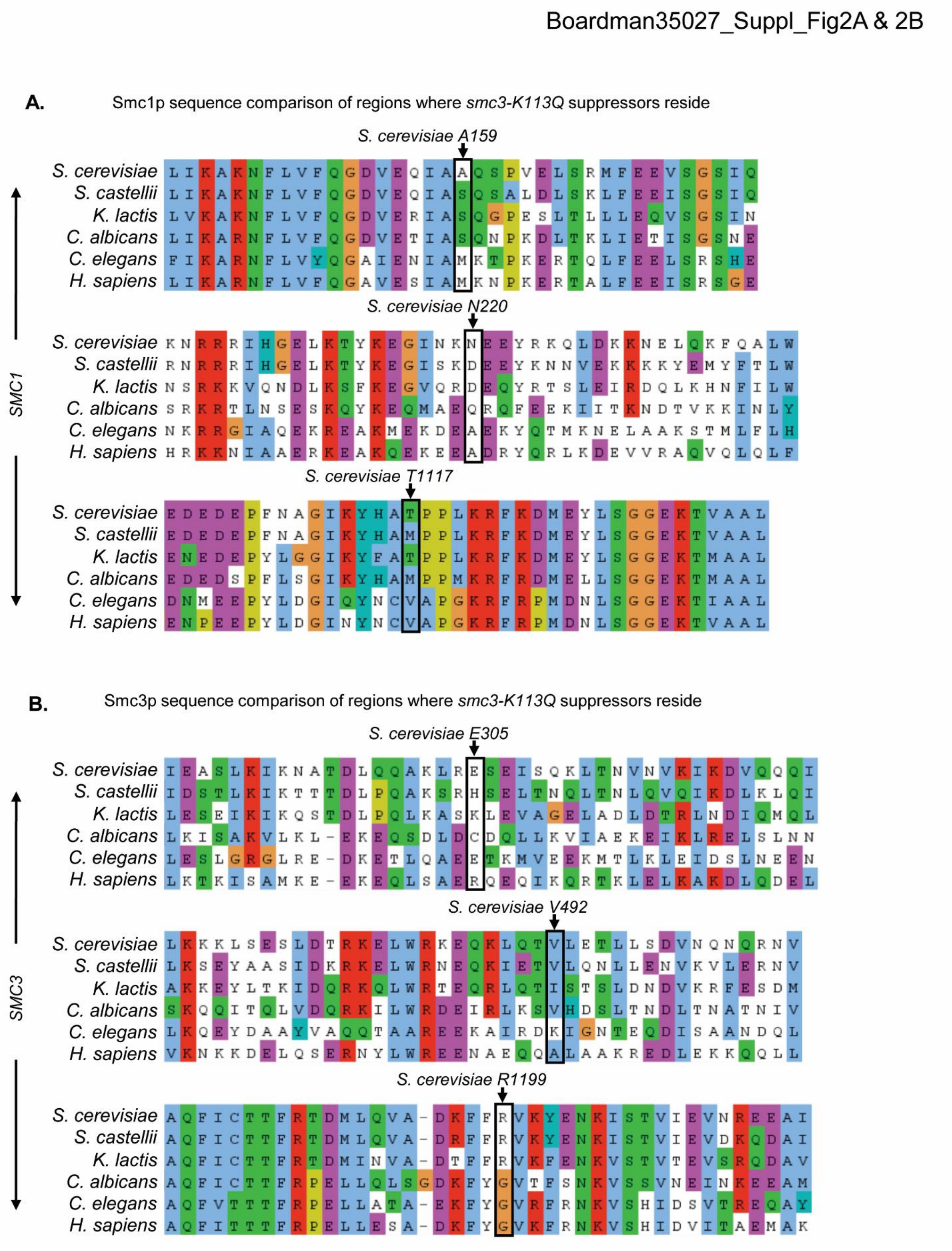
Boardman35027_Suppl_Fig2A & 2B
A.
Smc1p sequence comparison of regions where smc3-K113Q suppressors reside
S. cerevisiae A159
S. cerevisiae L I K A K N F L V F Q G D V E Q I A A Q S P V E L S R M F E E V S G S I Q
S. castellii L I K A K N F L V F Q G D V E Q I A S Q S A L D L S K L F E E I S G S I Q
K. lactis L V K A K N F L V F Q G D V E R I A S Q G P E S L T L L L E Q V S G S I N
C. albicans L I K A R N F L V F Q G D V E T I A S Q N P K D L T K L I E T I S G S N E
C. elegans F I K A R N F L V Y Q G A I E N I A M K T P K E R T Q L F E E L S R S H E
H. sapiens L I K A R N F L V F Q G A V E S I A M K N P K E R T A L F E E I S R S G E
S. cerevisiae N220
S. cerevisiae K N R R R I H G E L K T Y K E G I N K N E E Y R K Q L D K K N E L Q K F Q A L W
S. castellii R N R R R I H G E L K T Y K E G I S K D E E Y K N N V E K K K K Y E M Y F T L W
K. lactis N S R K K V Q N D L K S F K E G V Q R D E Q Y R T S L E I R D Q L K H N F I L W
C. albicans S R K R T L N S E S K Q Y K E Q M A E Q R Q F E E K I I T K N D T V K K I N L Y
C. elegans N K R R G I A Q E K R E A K M E K D E A E K Y Q T M K N E L A A K S T M L F L H
H. sapiens H R K K N I A A E R K E A K Q E K E E A D R Y Q R L K D E V V R A Q V Q L Q L F
SMC1
S. cerevisiae T1117
S. cerevisiae E D E D E P F N A G I K Y H A T P P L K R F K D M E Y L S G G E K T V A A L
S. castellii E D E D E P F N A G I K Y H A M P P L K R F K D M E Y L S G G E K T V A A L
K. lactis E N E D E P Y L G G I K Y F A T P P L K R F K D M E Y L S G G E K T M A A L
C. albicans E D E D S P F L S G I K Y H A M P P M K R F R D M E L L S G G E K T M A A L
C. elegans D N M E E P Y L D G I Q Y N C V A P G K R F R P M D N L S G G E K T I A A L
H. sapiens E N P E E P Y L D G I N Y N C V A P G K R F R P M D N L S G G E K T V A A L
B.
Smc3p sequence comparison of regions where smc3-K113Q suppressors reside
S. cerevisiae E305
S. cerevisiae I E A S L K I K N A T D L Q Q A K L R E S E I S Q K L T N V N V K I K D V Q Q Q I
S. castellii I D S T L K I K T T T D L P Q A K S R H S E L T N Q L T N L Q V Q I K D L K L Q I
K. lactis L E S E I K I K Q S T D L P Q L K A S K L E V A G E L A D L D T R L N D I Q M Q L
C. albicans L K I S A K V L K L - E K E Q S D L D C D Q L L K V I A E K E I K L R E L S L N N
C. elegans L E S L G R G L R E - D K E T L Q A E E T K M V E E K M T L K L E I D S L N E E N
H. sapiens L K T K I S A M K E - E K E Q L S A E R Q E Q I K Q R T K L E L K A K D L Q D E L
S. cerevisiae V492
S. cerevisiae L K K K L S E S L D T R K E L W R K E Q K L Q T V L E T L L S D V N Q N Q R N V
S. castellii L K S E Y A A S I D K R K E L W R N E Q K I E T V L Q N L L E N V K V L E R N V
K. lactis A K K E Y L T K I D Q R K Q L W R T E Q R L Q T I S T S L D N D V K R F E S D M
C. albicans S K Q Q I T Q L V D Q R K I L W R D E I R L K S V H D S L T N D L T N A T N I V
C. elegans L K Q E Y D A A Y V A Q Q T A A R E E K A I R D K I G N T E Q D I S A A N D Q L
H. sapiens V K N K K D E L Q S E R N Y L W R E E N A E Q Q A L A A K R E D L E K K Q Q L L
SMC3
S. cerevisiae R1199
S. cerevisiae A Q F I C T T F R T D M L Q V A - D K F F R V K Y E N K I S T V I E V N R E E A I
S. castellii A Q F I C T T F R T D M L Q V A - D R F F R V K Y E N K I S T V I E V D K Q D A I
K. lactis A Q F I C T T F R T D M I N V A - D T F F R V K F E N K V S T V T E V S R Q D A V
C. albicans A Q F I C T T F R P E L L Q L S G D K F Y G V T F S N K V S S V N E I N K E E A M
C. elegans A Q F V T T T F R P E L L A T A - E K F Y G V R F R N K V S H I D S V T R E Q A Y
H. sapiens A Q F I T T T F R P E L L E S A - D K F Y G V K F R N K V S H I D V I T A E M A K

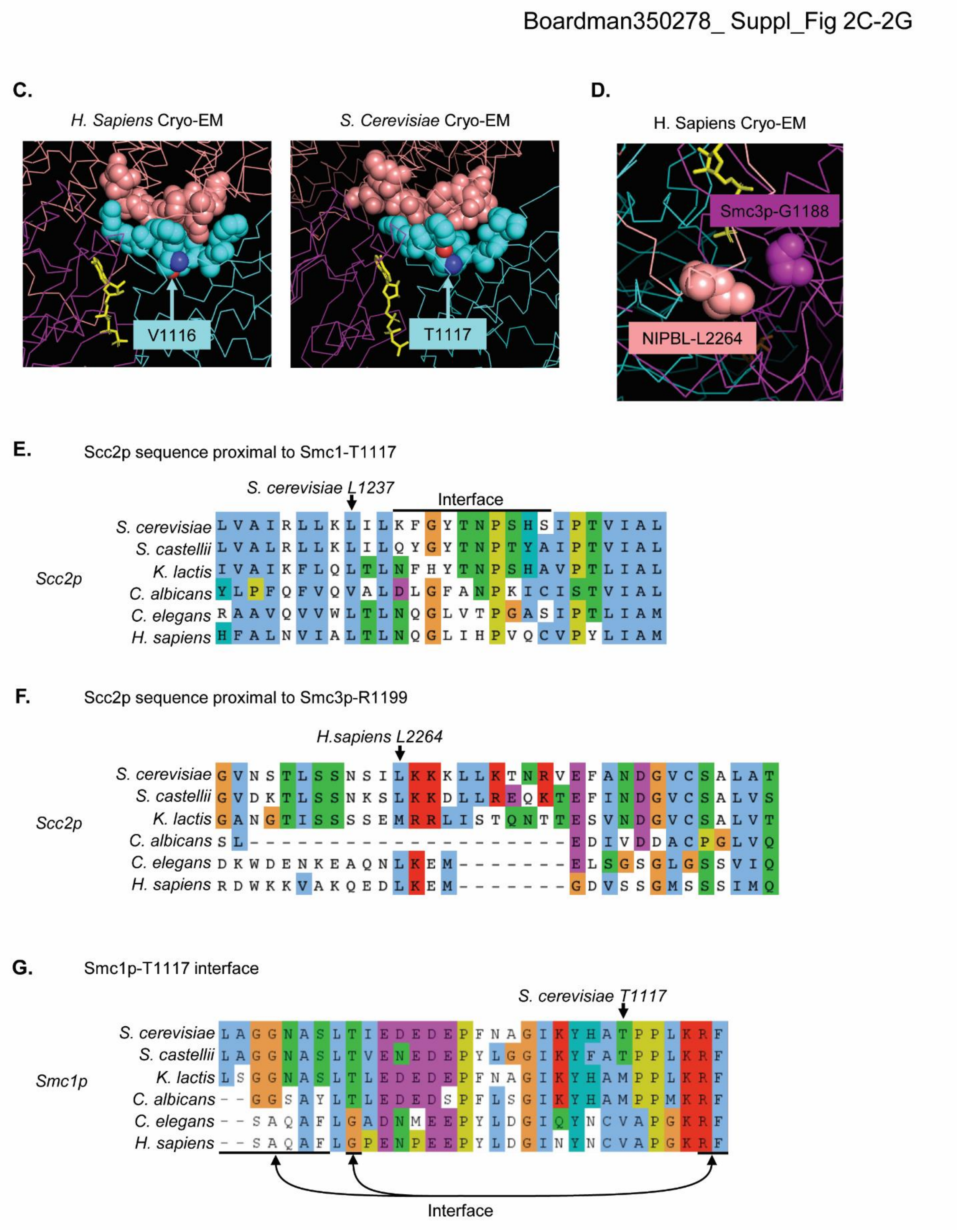
Boardman350278_ Suppl_Fig 2C-2G
C.
H. Sapiens Cryo-EM
S. Cerevisiae Cryo-EM
V1116
T1117
D.
H. Sapiens Cryo-EM
Smc3p-G1188
NIPBL-L2264
E.
Scc2p sequence proximal to Smc1-T1117
S. cerevisiae L1237
Interface
Scc2p
S. cerevisiae LVAIRLLKLILKFGYTNPSHSIPTVIAL
S. castellii LVALRLLKLILQYGYTNPTYAIPTVIAL
K. lactis IVAIKFLQLTLNFHYTNPSHAVPTLIAL
C. albicans YLPFQFVQVALDLGFANPKICISTVIAL
C. elegans RAAVQVVWLTLNQGLVTPGASIPTLIAM
H. sapiens HFALNVIALTLNQGLIHPVQCVPYLIAM
F.
Scc2p sequence proximal to Smc3p-R1199
H.sapiens L2264
Scc2p
S. cerevisiae GVNSTLSSNSILKKKLLKTNRVEFANDGVCSALAT
S. castellii GVDKTLSSNKSLKKDLLREQKTEFINDGVCSALVS
K. lactis GANGTISSSSEMRRLISTQNTTESVNDGVCSALVT
C. albicans SL--------------------EDIVDDACPGLVQ
C. elegans DKWDENKEAQNLKEM-------ELSGSGLGSSVIQ
H. sapiens RDWKKVAKQEDLKEM-------GDVSSGMSSSIMQ
G.
Smc1p-T1117 interface
S. cerevisiae T1117
Smc1p
S. cerevisiae LAGGNASLTIEDEDEPFNAGIKYHATPPLKRF
S. castellii LAGGNASLTVENEDEPYLGGIKYFATPPLKRF
K. lactis LSGGNASLTLEDEDEPFNAGIKYHAMPPLKRF
C. albicans --GGSAYLTLEDEDSPFLSGIKYHAMPPMKRF
C. elegans --SAQAFLGADNMEEPYLDGIQYNCVAPGKRF
H. sapiens --SAQAFLGPENPEEPYLDGINYNCVAPGKRF
Interface

Figure 2S

(A) Amino acid sequence alignment of Smc1p orthologs indicating the positions of the three suppressor *S. cerevisiae* residues: A159, N220 and T1117.

(B) Amino acid sequence alignment of Smc3p orthologs indicating the positions of the three suppressor *S. cerevisiae* residues: E305, V492 and R1199.

(C) Cryo-EM structure of *H. sapiens* (left) (PDB ID: 6WG3, Shi et al. 2020) and *S. cerevisiae* (right) (PDB ID: 6ZZ6, Collier et al. 2020) indicating Smc1p (cyan) and Scc2p (salmon), and ATP (yellow). Multiple residues are depicted as spheres to illustrate where the Smc1p-T1117 region forms an interface with Scc2p.

(D) *H. sapiens* cryo-EM (PDB ID: 6WG3, Shi et al. 2020) illustrating that Smc3p-G1188 (magenta) interfaces with NIPBL (Salmon) indicated as spheres. ATP is yellow.

(E) Amino acid sequence alignment of Scc2p orthologs showing the region which interfaces with *S. cerevisiae* Smc1p-T1117.

(F) Amino acid sequence alignment of NIPBL/Scc2p orthologs showing the *H. sapiens* Scc2p region that interfaces with Smc3p-G1188 (Smc3p-R1199 in *S. cerevisiae*).

(G) Amino acid sequence alignment of Smc1 orthologs showing the Smc1p-T1117 region. *S. cerevisiae* Smc1p residues which interfaces with Scc2p are indicated.

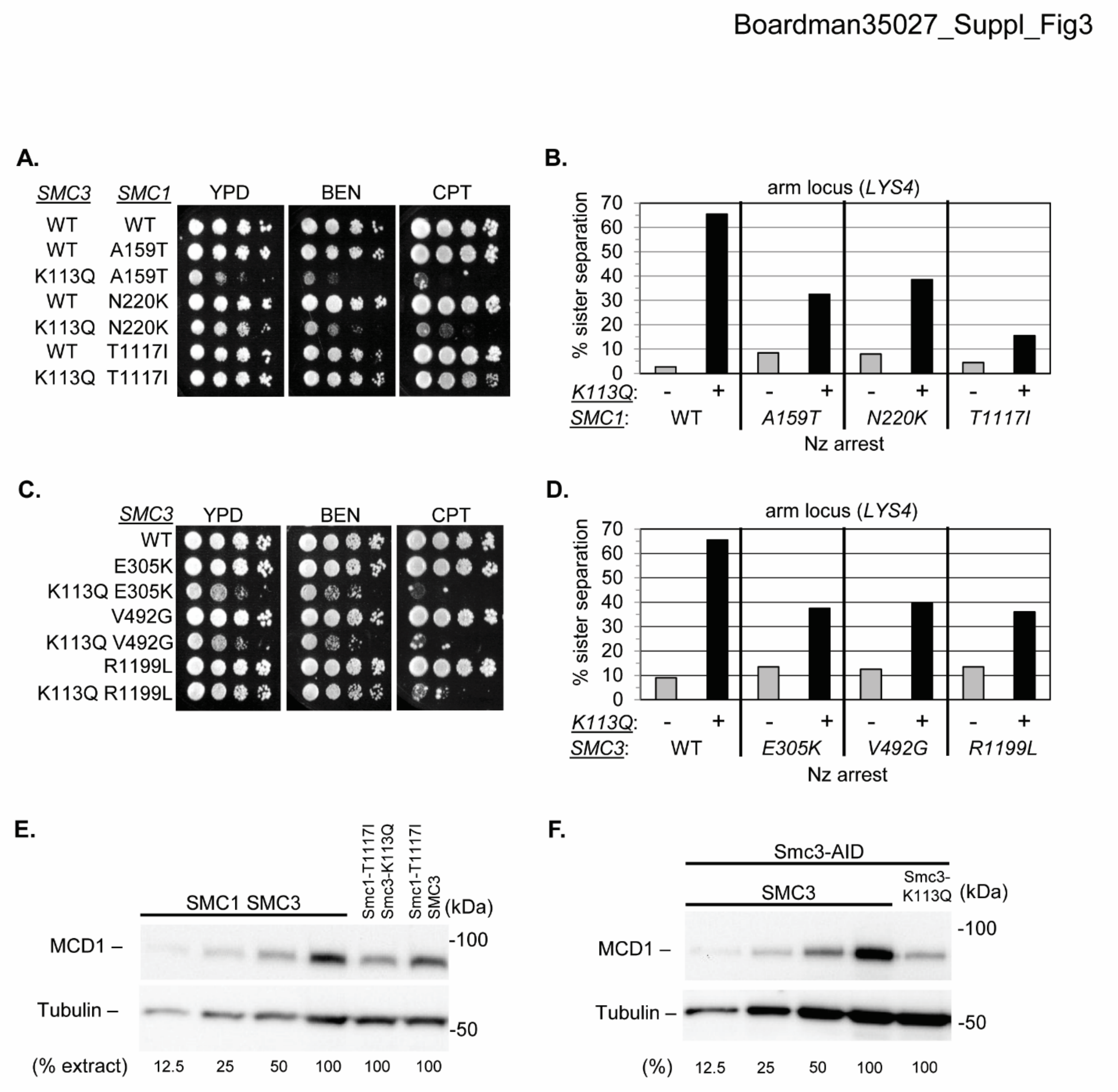

Figure 3S

smc1-T1117I is the most robust of the six suppressors of *smc3-K113Q* inviability and cohesion defects.

(A-B) Comparison of the three *SMC1* suppressor mutations shows only *smc1-T1117I* restores nearly wild-type function to *smc3-K113Q* cohesin.

(A) The *smc1-T1117I smc3-K113Q* double mutant exhibits growth and drug resistance similar to the wild-type whereas *smc1-A159T smc3-K113Q* and *smc1-N220K smc3-K113Q* exhibit growth

defects and drug sensitivity. Haploid wild-type (WT) (VG4012-2C), *smc1-A159T* (VG4004-7A), *smc3-K113Q smc1-A159T* (VG4008-2B), *smc1-N220K* (VG4005-10A), *smc3-K113Q smc1-N220K* (VG4009-6B), *smc1-T1117I* (VG4006-13A) and *smc3-K113Q smc1-T1117I* (VG4010-8B) strains were grown and diluted as described in Figure 1C then plated on YPD alone or containing benomyl [BEN] (10mg/ml) or camptothecin [CPT] (15mg/ml) and incubated for 3d at 23°C, 4d at 23°C or 3d at 30°C respectively.

(B) *smc1-T1117I* strongly suppresses cohesion defect of the *smc3-K113Q* mutant. Haploid strains in (A) were synchronously arrested in mid-M phase and cohesion loss assessed at a chromosome arm site (*LYS4*) as described in Figure 1D.

(C-D) Comparison of the three *smc3* suppressors of *smc3-K113Q* shows they all exhibit some defects.

(C) The three *smc3* suppressor *smc3-K113Q* double mutants exhibit growth defects and drug sensitivity. Haploid wild-type (VG3986-2A), *smc3-E305K* (VG3997-4C), *smc3-K113Q smc3-E305K* (VG3998-5B), r*smc3-V492G* (VG4000-1A), *smc3-K113Q smc3-V492G* (VG4001-1B), *smc3-R1199L* (VG4002-4A), *smc3-K113Q smc3-R1199L* (VG4003-4B), were grown and plated as described in (A).

(D) The three *smc3* suppressors partially suppress the cohesion defect of the *smc3-K113Q* mutant. Haploid strains in (C) were synchronously arrested in mid-M phase and cohesion loss assessed at a chromosome arm site (*LYS4*) as described in Figure 1D.

(E) Mcd1p levels are restored to approximately 50% of wildtype in the *smc1-T1117I smc3-K113Q* double mutant. Protein extracts (TCA lysed) from 2 OD aliquots of mid-M phase arrested cells from (C) were analyzed by Western blot. Mcd1p protein levels were monitored using rabbit antibodies against Mcd1p (αMCD1) and rabbit antibodies against tubulin (αTUB2) served as a loading control.

(F) Mcd1p levels in mid-M phase cells are reduced when acetyl-mimic mutant *smc3-K113Q* is the sole Smc3p present. Protein extracts (TCA lysed) from 2 OD aliquots of mid-M phase

arrested cells from Figure 3B were analyzed by Western blot. Mcd1p protein levels were monitored using rabbit antibodies against Mcd1p (αMCD1) and rabbit antibodies against tubulin (αTUB2) served as a loading control.

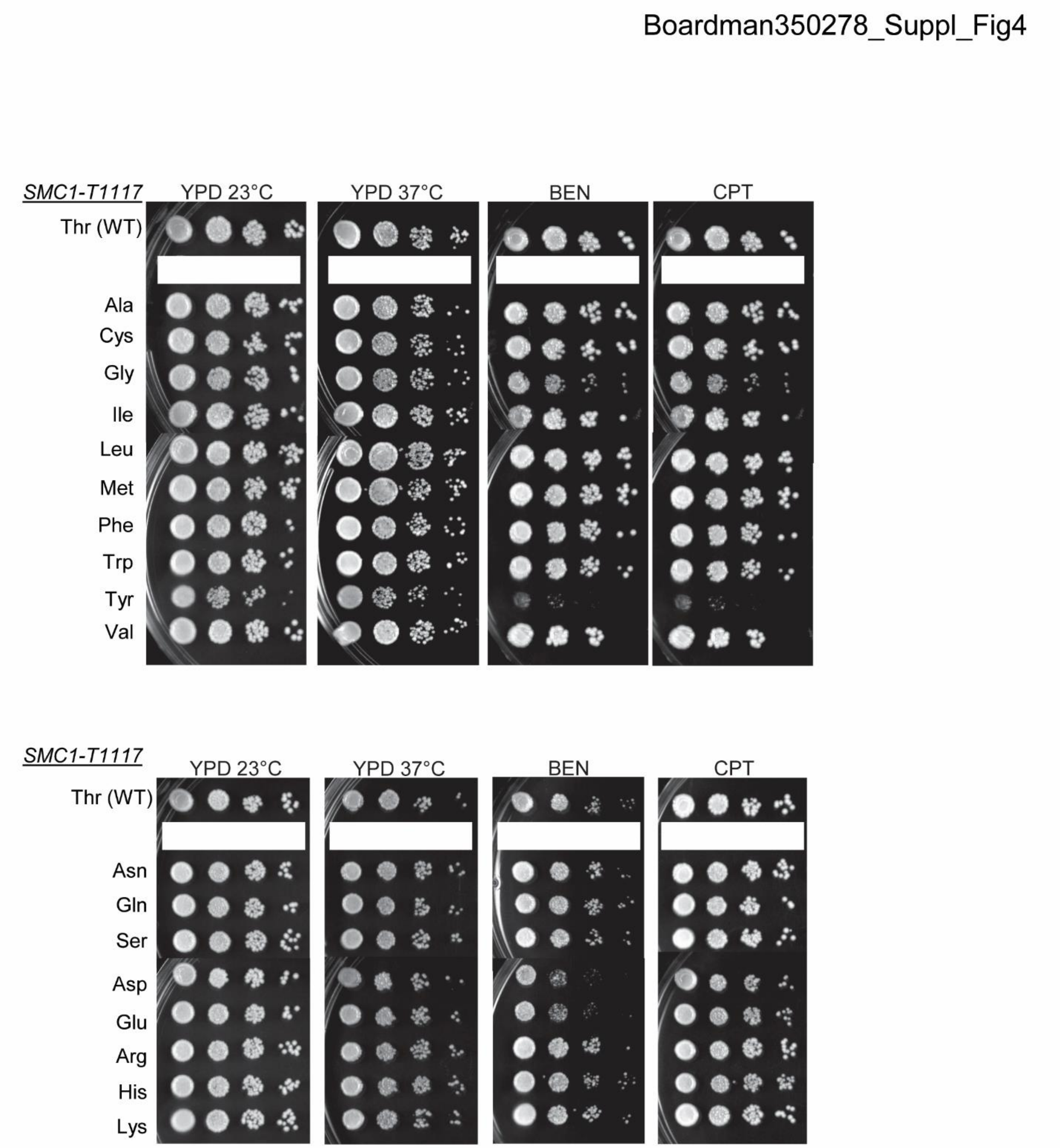

Figure 4S

Random mutagenesis of the *smc1-T1117* residue in a wild-type background reveals that 19 of the 20 amino acids can support cell viability. CRISPR was used to insert random substitutions of smc1-T1117 residues in a haploid wild-type strain (VG3620-4C) as described in Materials and Methods. Transformant colonies were PCR sequenced to determine which *smc1-T1117*

substitutions are viable. 19 amino acids substitutions were identified but proline could not be found. A specific CRISPR repair to generate Smc1-T1117P confirmed a proline substitution cannot support viability. Single colonies containing each different amino acid were grown and diluted as described in Figure 1C then plated on YPD and incubated 3d at 23°C or 2d at 37°C or on YPD containing 12.5mg/ml benomyl [BEN] or (25mg/ml camptothecin [CPT] and incubated for 4d at 23°C. All T1117 substitutions grew well at 23°C and 37°C and most were drug resistant. The tyrosine substitution was sensitive to both BEN and CPT whereas aspartic acid and glutamic acid were only BEN sensitive. Plates were electronically rearranged for ease of display.

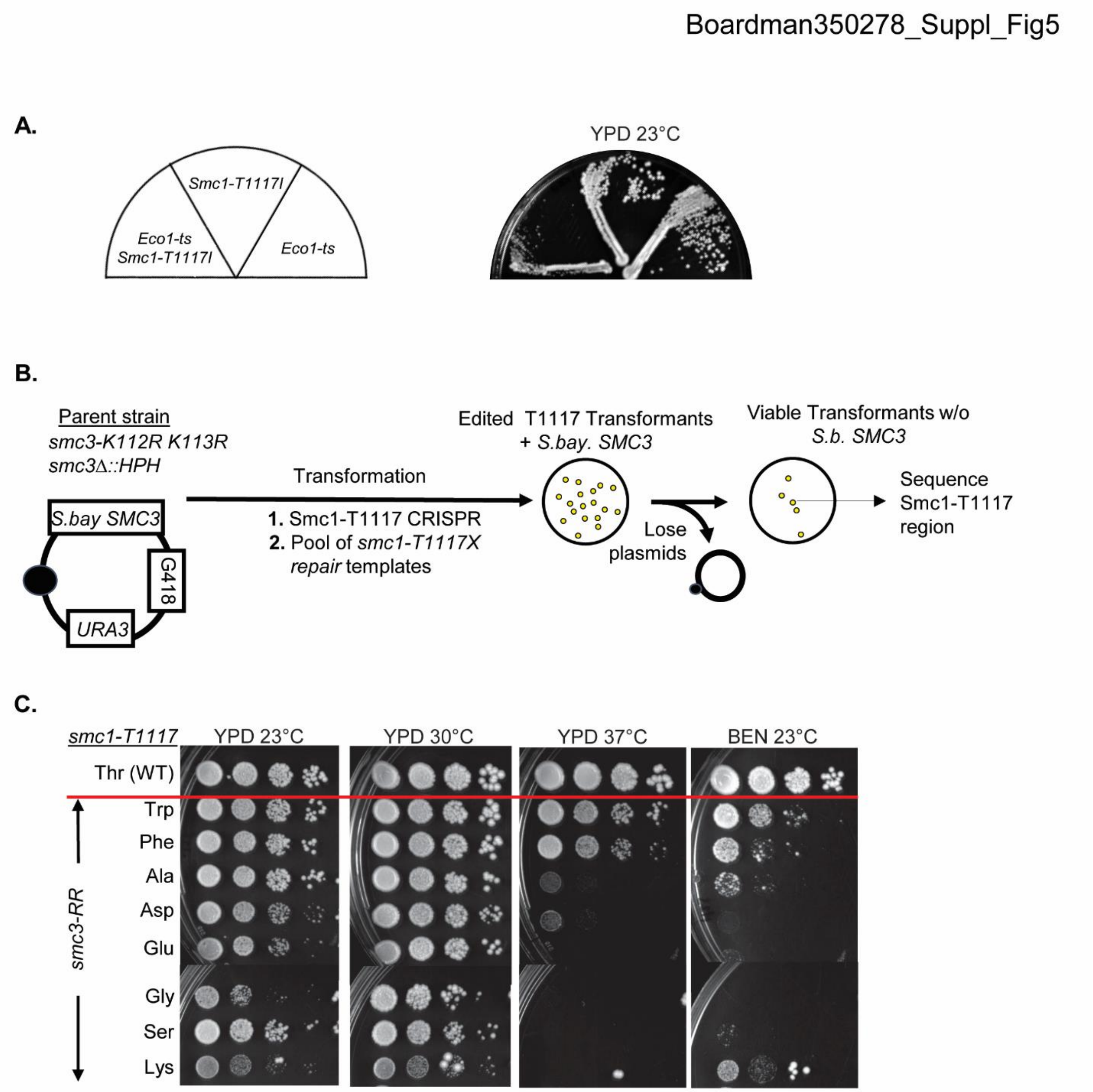

Figure 5S

Substitutions at *smc1-T1117* can suppress an Smc3 acetyl-null (*K112R K113R*).

(A) *smc1-T1117I* hinders the growth of *eco1-ts (ctf7-203)* at 23°C. An *eco1-203* strain (VG3223-12B), *smc1-T1117I eco1-203 strain* and an *smc1-T1117I* (VG4006-13A) strain were dilution streaked on YPD plates and grown at the 23°C for 4 days.

(B) Schematic of the screen to assess whether any residues at *smc1-T1117* can suppress *smc3-K112R K113R* (RR). Haploid strain (VG4144-5C), containing *smc3-K112R, K113R* (RR)

as the sole *S. cerevisiae SMC3* and *S. Bayanus SMC3* on a *CEN URA3 G418* plasmid (pFC3). CRISPR was used to insert random residues at *S. cerevisiae smc1-T1117*. Viable transformants bearing *smc3-RR* as the sole Smc3p in cells were PCR sequenced to identify the *smc1-T1117* suppressor residue.

(C) *smc1-T1117* residues that suppress *smc3-K112R, K113R* (RR) lethality exhibit different growth and drug sensitivity. Haploid wild-type (VG3620-4C) and various *smc3-RR* strains bearing suppressor mutations at the T1117 residue, *T1117W* (Trp), *T1117F* (Phe), *T1117A* (Ala), *T1117E* (Asp), *T1117E* (Glu), *T1117G* (Gly), *T1117S* (Ser) and *T1117K* (Lys), were grown to saturation at 23°C, and then plated at 10-fold serial dilution on YPD and incubated at 23°C 4d, 30°C 3d or 37°C 3d or on YPD containing 10mg/ml benomyl and incubated 23°C 5d (BEN 23°C). Plates were electronically rearranged for ease of display.

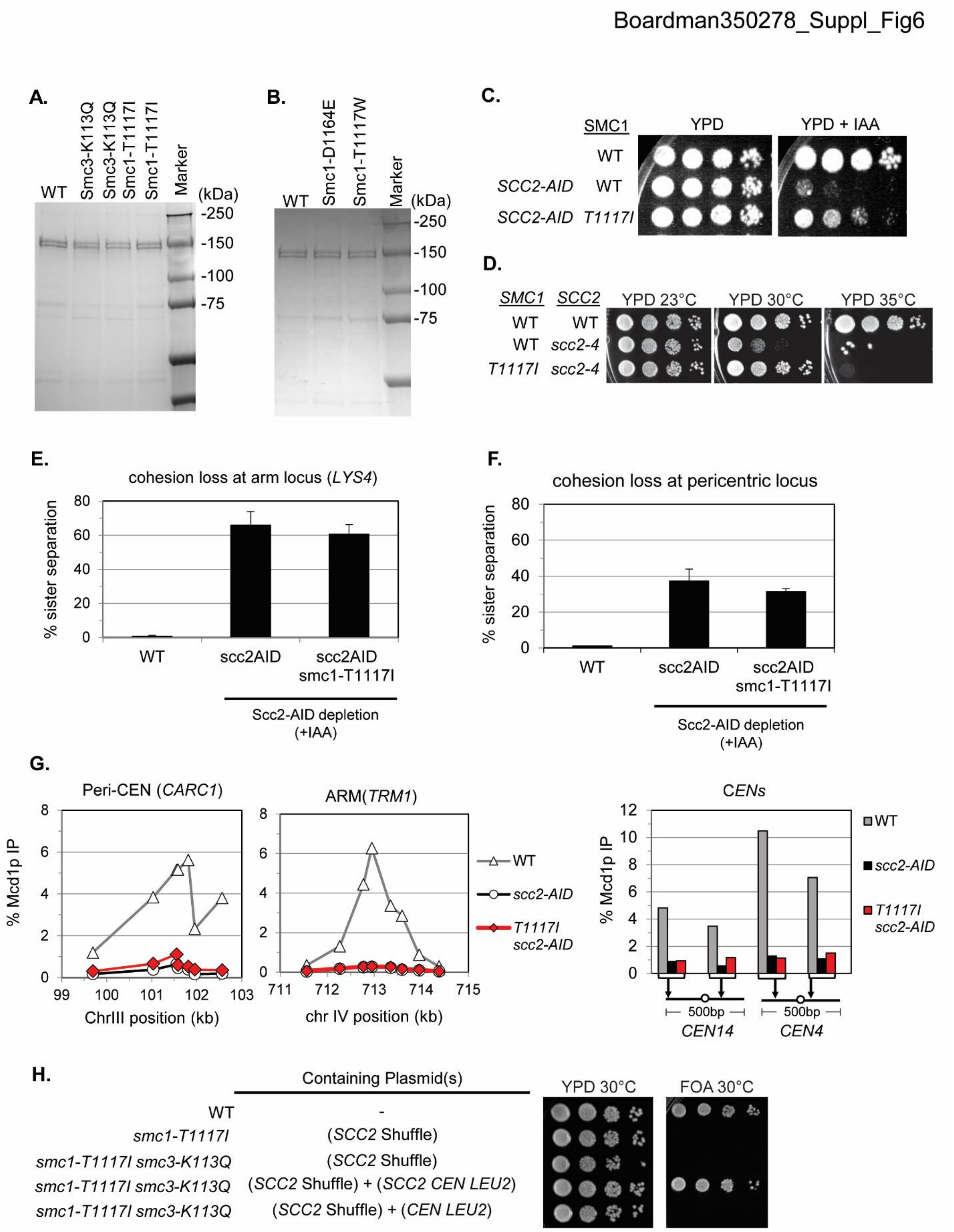
Boardman350278_Suppl_Fig6
A.
WT
Smc3-K113Q
Smc3-K113Q
Smc1-T1117I
Smc1-T1117I
Marker
(kDa)
-250
-150
-100
-75
B.
WT
Smc1-D1164E
Smc1-T1117W
Marker
(kDa)
-250
-150
-100
-75
C.
SMC1
YPD
YPD + IAA
WT
SCC2-AID WT
SCC2-AID T1117I
D.
SMC1 SCC2
YPD 23°C
YPD 30°C
YPD 35°C
WT WT
WT scc2-4
T1117I scc2-4
E.
cohesion loss at arm locus (LYS4)
% sister separation
WT
scc2AID
scc2AID smc1-T1117I
Scc2-AID depletion (+IAA)
F.
cohesion loss at pericentric locus
% sister separation
WT
scc2AID
scc2AID smc1-T1117I
Scc2-AID depletion (+IAA)
G.
Peri-CEN (CARC1)
% Mcd1p IP
ChrIII position (kb)
ARM(TRM1)
chr IV position (kb)
WT
scc2-AID
T1117I scc2-AID
CENs
% Mcd1p IP
WT
scc2-AID
T1117I scc2-AID
500bp
500bp
CEN14
CEN4
H.
Containing Plasmid(s)
WT -
smc1-T1117I (SCC2 Shuffle)
smc1-T1117I smc3-K113Q (SCC2 Shuffle)
smc1-T1117I smc3-K113Q (SCC2 Shuffle) + (SCC2 CEN LEU2)
smc1-T1117I smc3-K113Q (SCC2 Shuffle) + (CEN LEU2)
YPD 30°C
FOA 30°C

Figure 6S.

(A) Coomassie stain of purified and normalized concentration of wild-type (WT) and mutant cohesin complexes presented in Figure 7A.

(B) Coomassie stain of purified and normalized concentration of wild-type (WT) and mutant cohesin complexes presented in Figure 7B.

(C) *smc1-T1117I* weakly compensates for reduced Scc2p but does not replace Scc2 function.

smc1-T1117I suppresses the inviability of *scc2-AID* depletion. Haploid wild-type (VG3620-4C), *scc2-AID* (VG3630-7A) and *smc1-T1117I scc2-AID* (VG4146-1C) strains were grown and diluted as described in Figure 1C then plated on YPD alone or containing IAA then incubated at 23°C for 3d.

(D) *smc1-T1117I* cannot suppress a temperature sensitive Scc2p. Haploid wild-type (VG3620-4C), *scc2-4* (VG3308-9A) and double mutant *smc1-T1117I scc2-4* (VG4162-2A) were grown at 23°C to saturation, plated at 10-fold serial dilutions on YPD and incubated at 23°C or 35°C for 3 days.

(E-F) *smc1-T1117I* fails to suppress the cohesion defect of *scc2-AID* depleted cells.

(E) Cohesion loss at a chromosomal arm locus (*LYS4*). Strains in (A) were in synchronously arrested mid-M phase under conditions that deplete Scc2p-AID and cohesion loss at *LYS4* as described in Figure 1D.

(F) Cohesion loss at a *CEN*-proximal locus. Haploid wild-type (TE631), *scc2-AID* (VG4163-3A) and *smc1-T1117I scc2-AID* (VG4164-4B) were synchronously arrested in mid-M phase and cohesion loss at a *CEN*-proximal locus assessed as described in Figure 1D.

(G) *smc1-T1117I* requires Scc2p to load cohesin on chromosomes. Aliquots of mid-M phase arrested cells from (E) were fixed and processed for ChIP, then the level of cohesin bound to

chromosomes determined as described in Figure 1E. Left panel is chromosome IV arm *CAR* region (*TRM1*), middle panel is chromosome III peri-centric region (*CARC1*), and right panel are regions immediately adjacent to *CEN14* and *CEN4*.

(H) *smc1-T1117I* mutation is unable to bypass the need for *SCC2* in either a wild-type background or *smc3-K113Q* background. Plasmid shuffle assay with wild-type (VG3620-4C), *smc1-T1117I scc2*Δ + pVG587 (*SCC2* shuffle plasmid) (VG4210-1D), *smc1-T1117I smc3-K113Q scc2*Δ + pVG587 (VG4215-1B), *smc1-T1117I smc3-K113Q scc2*Δ + pVG587 & p3555 (SCC2 CEN LEU2) (VG4215-1B + p3555), and *smc1-T1117I smc3-K113*Q *scc2*Δ + pVG587 & pRS315 (CEN LEU2) (VG4215-1B + pRS315). Strains were grown and diluted as in Figure 1C then plated on YPD and 5-FOA media and incubated for 2 days at 30°C.
