# Supplementary material for: A model for Scc2p stimulation of cohesin's ATPase and its inhibition by acetylation of Smc3p
Source: Genes Dev. 2023 Apr 1;37(7-8):277–90. doi: 10.1101/gad.350278.122 (PMC10153460; doi:10.1101/gad.350278.122)
Supplement: Supplemental Material [file supp_gad.350278.122_Supplemental_Table_1.pdf]

Supplemental Table 1 – Strain Table

Strain	Genotype	Background
VG3919-3C	MATa SMC3-LEU2:leu2-3,112 smc3-N607-3V5-AID TIR1-CaTRP1 LacO-NAT::lys4 GFPLacI-HIS3:his3-11,15 ura3-52 bar1 GAL+	A346A
VG3981-6B	MATa SMC3-K113Q-LEU2:leu2-3,112 smc3-N607-3V5-AID TIR1-CaTRP1<br>LacO-NAT::lys4 GFPLacI-HIS3:his3-11,15 ura3-52 bar1 GAL+	A346A
VG4052-3A	MATa pGAL-SCC2/4-URA3:ura3-52 TIR1-CaTRP1 smc3-K113Q-LEU2:leu2-3,112 smc3-N607-3V5-AID LacO-NAT::lys4 GFPLacI-HIS3:his3-11,15 bar1 GAL+	A346A
VG3651-3D	MATa SMC3-N607-3V5-AID TIR1-CgTRP1 LacO-NAT::lys4 leu2-3,112 ura3-52 GFPLacI-HIS3:his3-11,15 bar1 GAL+	A346A
JL17A	MATa SMC3-LEU2:leu2-3,112 smc3-N607-3V5-AID TIR1-CaTRP1 LacO:10kb-CEN4-NAT GFPLacI-HIS3:his3-11,15 ura3-52 bar1 GAL+	A346A
JL16A	MATa SMC3-N607-3V5-AID TIR1-CgTRP1 LacO:10kb-CEN4-NAT leu2-3,112 ura3-52 GFPLacI-HIS3:his3-11,15 bar1 GAL+	A346A
JL14A	MATa SMC3-K113Q-LEU2:leu2-3,112 smc3-N607-3V5-AID TIR1-CaTRP1 LacO:10kb-CEN4-NAT GFPLacI-HIS3:his3-11,15 URA3:ura3-52 bar1 GAL+	A346A
JL15A	MATa pGAL-SCC2/4-URA3:ura3-52 TIR1-CaTRP1 smc3-K113Q-LEU2:leu2-3,112 smc3-N607-3V5-AID LacO:10kb-CEN4-NAT GFPLacI-HIS3:his3-11,15 bar1 GAL+	A346A
KB61A	MATa TIR1-CgTRP1 LacO-NAT::lys4 GFPLacI-HIS3:his3-11,15 URA3:ura3-52 leu2-3,112 bar1 GAL+	A346A
VG3633-2D	MATa ECO1-3V5-AID2-G418 TIR1-CaTRP1 LacO-NAT::lys4 leu2-3,112 GAL+ pHIS3-GFPLacI-HIS3:his3-11,15 bar1 ura3-52	A346A
KB50A	MATa ECO1-3V5-AID2-G418 TIR1-CaTRP1 LacO-NAT::lys4 pGAL-SCC2,SCC4-LEU2:leu2-3,112 GAL+ pHIS3-GFPLacI-HIS3:his3-11,15 bar1 ura3-52	A346A
VG3862-1A	MATa PDS5-3V5-AID2F:G418 TIR1-CaTRP1 LacO-NAT::lys4 GFPLacI-HIS3:his3-11,15 leu2-3,112 ura3-52 bar1 GAL+	A346A
KB46A	MATa PDS5-3V5-AID2F:G418 TIR1-CaTRP1 LacO-NAT::lys4 GFPLacI-HIS3:his3-11,15 leu2-3,112 pGAL-SCC2,SCC4-URA3:ura3-52 bar1 GAL+	A346A
KB47A	MATa SMC3-N607-3V5-AID TIR1-CgTRP1 LacO-NAT::lys4 leu2-3,112 URA3-pGAL1,10-SCC2, SCC4::ura3-52 GFPLacI-HIS3:his3-11,15 bar1 GAL+	A346A

VG3969-14C	MATa smc3Δ::HPH smc3-K113Q-LEU2:leu2-3,112 LacO-NAT::lys4 GFPLacI-TRP1:his3-11,15 trp1-1 ura3-52 bar1 GAL+ w/pFC3	A346A
VG4012-2C	MATa SMC1-LEU2:leu2-3,112 smc1Δ::HPH LacO-NAT::lys4 GFPLacI-HIS3:his3-11,15 trp1-1 ura3-52 bar1 GAL+	A346A
VG4005-10A	MATa smc1-N220K-LEU2:leu2-3,112 smc1Δ::HPH LacO-NAT::lys4 GFPLacI-HIS3:his3-11,15 trp1-1 ura3-52 bar1 GAL+	A346A
VG4009-6B	MATa smc3-K113Q smc1-N220K-LEU2:leu2-3,112 smc1Δ::HPH LacO-NAT::lys4 GFPLacI-HIS3:his3-11,15 trp1-1 ura3-52 bar1 GAL+	A346A
VG4006-13A	MATa smc1-T1117I-LEU2:leu2-3,112 smc1Δ::HPH LacO-NAT::lys4 GFPLacI-HIS3:his3-11,15 trp1-1 ura3-52 bar1 GAL+	A346A
VG4010-8B	MATa smc3-K113Q smc1-T1117I-LEU2:leu2-3,112 smc1Δ::HPH LacO-NAT::lys4 GFPLacI-HIS3:his3-11,15 trp1-1 ura3-52 bar1 GAL+	A346A
3620-4C	MATa TIR1-CgTRP1 LacO-NAT::lys4 GFPLacI-HIS3:his3-11,15 leu2-3,112 ura3-52 bar1 GAL+	A346A
VG4004-7A	MATa smc1-A159T-LEU2:leu2-3,112 smc1Δ::HPH LacO-NAT::lys4 GFPLacI-HIS3:his3-11,15 trp1-1 ura3-52 bar1 GAL+	A346A
VG4008-2B	MATa smc3-K113Q smc1-A159T-LEU2:leu2-3,112 smc1Δ::HPH LacO-NAT::lys4 GFPLacI-HIS3:his3-11,15 trp1-1 ura3-52 bar1 GAL+	A346A
VG3986-2A	MATa SMC3-LEU2:leu2-3,112 smc3Δ::HPH LacO-NAT::lys4 GFPLacI-TRP1:his3-11,15 trp1-1 ura3-52 bar1 GAL+	A346A
VG3997-4C	MATa smc3Δ::HPH smc3-E305K-LEU2:leu2-3,112 LacO-NAT::lys4 GFPLacI-TRP1:his3-11,15 trp1-1 ura3-52 bar1 GAL+	A346A
VG3998-5B	MATa smc3Δ::HPH smc3-K113Q,E305K-LEU2:leu2-3,112 LacO-NAT::lys4 GFPLacI-TRP1:his3-11,15 trp1-1 ura3-52 bar1 GAL+	A346A
VG4000-1A	MATa smc3-V492G-LEU2:leu2-3,112 smc3Δ::HPH LacO-NAT::lys4 GFPLacI-TRP1:his3-11,15 trp1-1 ura3-52 bar1 GAL+	A346A
VG4001-1B	MATa smc3-K113Q,V492G-LEU2:leu2-3,112 smc3Δ::HPH LacO-NAT::lys4 GFPLacI-TRP1:his3-11,15 trp1-1 ura3-52 bar1 GAL+	A346A
VG4002-4A	MATa smc3-R1199L-LEU2:leu2-3,112 smc3Δ::HPH LacO-NAT::lys4 GFPLacI-TRP1:his3-11,15 trp1-1 ura3-52 bar1 GAL+	A346A
VG4003-4B	MATa smc3-K113Q,R1199L-LEU2:leu2-3,112 smc3Δ::HPH LacO-NAT::lys4 GFPLacI-TRP1:his3-11,15 trp1-1 ura3-52 bar1 GAL+	A346A
VG3223-12B	MATa ctf7-203 LacO-NAT::lys4 pHIS3-GFPLacI-HIS3:his3-11,15 GAL+ trp1-1 leu2-3,112 ura3-52 bar1	A346A
KB88	MATa ctf7-203 smc1-T1117I LacO-NAT::lys4 pHIS3-GFPLacI-HIS3:his3-11,15 GAL+ trp1-1 leu2-3,112 ura3-52 bar1	A346A

VG4147-14C	MATa smc3-K113Q smc1-T1117V-LEU2:leu2-3,112 smc1Δ::HPH LacO-NAT::lys4 GFPLacI-HIS3:his3-11,15 trp1-1 ura3-52 bar1 GAL+	A346A
VG3633-3D	MATa ECO1-3V5-AID2-G418 TIR1-CaTRP1 LacO-NAT::lys4 leu2-3,112 GAL+ pHIS3-GFPLacI-HIS3:his3-11,15 bar1 ura3-52	A346A
KB118E	MATa smc1-T1117I ECO1-3V5-AID2-G418 TIR1-CaTRP1 LacO-NAT::lys4 leu2-3,112 GAL+pHIS3-GFPLacI-HIS3:his3-11,15 bar1 ura3-52	A346A
JL11A	MATa smc1-T1117I TIR1-CgTRP1 LacO-NAT::lys4 GFPLacI-HIS3:his3-11,15 leu2-3,112 ura3-52 bar1 GAL+	A346A
VG3620-4C Ala	MATa smc1-T1117A TIR1-CgTRP1 LacO-NAT::lys4 GFPLacI-HIS3:his3-11,15 leu2-3,112 ura3-52 bar1 GAL+	A346A
VG3620-4C Cys	MATa smc1-T1117C TIR1-CgTRP1 LacO-NAT::lys4 GFPLacI-HIS3:his3-11,15 leu2-3,112 ura3-52 bar1 GAL+	A346A
VG3620-4C Gly	MATa smc1-T1117G TIR1-CgTRP1 LacO-NAT::lys4 GFPLacI-HIS3:his3-11,15 leu2-3,112 ura3-52 bar1 GAL+	A346A
VG3620-4C Ile	MATa smc1-T1117I TIR1-CgTRP1 LacO-NAT::lys4 GFPLacI-HIS3:his3-11,15 leu2-3,112 ura3-52 bar1 GAL+	A346A
VG3620-4C Leu	MATa smc1-T1117L TIR1-CgTRP1 LacO-NAT::lys4 GFPLacI-HIS3:his3-11,15 leu2-3,112 ura3-52 bar1 GAL+	A346A
VG3620-4C Met	MATa smc1-T1117M TIR1-CgTRP1 LacO-NAT::lys4 GFPLacI-HIS3:his3-11,15 leu2-3,112 ura3-52 bar1 GAL+	A346A
VG3620-4C Phe	MATa smc1-T1117F TIR1-CgTRP1 LacO-NAT::lys4 GFPLacI-HIS3:his3-11,15 leu2-3,112 ura3-52 bar1 GAL+	A346A
VG3620-4C Trp	MATa smc1-T1117W TIR1-CgTRP1 LacO-NAT::lys4 GFPLacI-HIS3:his3-11,15 leu2-3,112 ura3-52 bar1 GAL+	A346A
VG3620-4C Tyr	MATa smc1-T1117Y TIR1-CgTRP1 LacO-NAT::lys4 GFPLacI-HIS3:his3-11,15 leu2-3,112 ura3-52 bar1 GAL+	A346A
VG3620-4C Val	MATa smc1-T1117V TIR1-CgTRP1 LacO-NAT::lys4 GFPLacI-HIS3:his3-11,15 leu2-3,112 ura3-52 bar1 GAL+	A346A
VG3620-4C Asn	MATa smc1-T1117N TIR1-CgTRP1 LacO-NAT::lys4 GFPLacI-HIS3:his3-11,15 leu2-3,112 ura3-52 bar1 GAL+	A346A
VG3620-4C Gln	MATa smc1-T1117Q TIR1-CgTRP1 LacO-NAT::lys4 GFPLacI-HIS3:his3-11,15 leu2-3,112 ura3-52 bar1 GAL+	A346A
VG3620-4C Ser	MATa smc1-T1117S TIR1-CgTRP1 LacO-NAT::lys4 GFPLacI-HIS3:his3-11,15 leu2-3,112 ura3-52 bar1 GAL+	A346A
VG3620-4C Asp	MATa smc1-T1117D TIR1-CgTRP1 LacO-NAT::lys4 GFPLacI-HIS3:his3-11,15 leu2-3,112 ura3-52 bar1 GAL+	A346A

VG3620-4C Glu	MATa smc1-T1117E TIR1-CgTRP1 LacO-NAT::lys4 GFPLacI-HIS3:his3-11,15 leu2-3,112 ura3-52 bar1 GAL+	A346A
VG3620-4C Arg	MATa smc1-T1117R TIR1-CgTRP1 LacO-NAT::lys4 GFPLacI-HIS3:his3-11,15 leu2-3,112 ura3-52 bar1 GAL+	A346A
VG3620-4C His	MATa smc1-T1117H TIR1-CgTRP1 LacO-NAT::lys4 GFPLacI-HIS3:his3-11,15 leu2-3,112 ura3-52 bar1 GAL+	A346A
VG3620-4C Lys	MATa smc1-T1117K TIR1-CgTRP1 LacO-NAT::lys4 GFPLacI-HIS3:his3-11,15 leu2-3,112 ura3-52 bar1 GAL+	A346A
VG3630-7A	MATa SCC2-3V5-AID2-G418 TIR1-CgTRP1 LacO-NAT::lys4 leu2-3,112 GAL+<br>GFPLacI-HIS3:his3-11,15 bar1 ura3-52	A346A
VG4146-1C	MATa smc1-T1117I SCC2-3V5-AID2-G418 TIR1-CgTRP1 LacO-NAT::lys4<br>GFPLacI-HIS3:his3-11,15 leu2-3,112 ura3-52 bar1 GAL+	A346A
TE631	MATa LacO-NAT:10KB-CEN4 TIR1-CaTRP1 leu2-3,112 GFPLacI-HIS3:his3-11,15 ura3-52 bar1 GAL+	A346A
VG4163-3A	MATa SCC2-3V5-AID2-G418 TIR1-CgTRP1 LacO-NAT:10kb-CEN4 leu2-3,112<br>GFPLacI-HIS3:his3-11,15 ura3-52 bar1 GAL+	A346A
VG4164-4B	MATa smc1-T1117I SCC2-3V5-AID2-G418 TIR1-CgTRP1 LacO-NAT:10kb-CEN4<br>GFPLacI-HIS3:his3-11,15 leu2-3,112 ura3-52 bar1 GAL+	A346A
VG3308-9A	MATa scc2-4 trp1-1 leu2-3,112 ura3-52 GFPLacI-HIS3:his3-11,15 bar1 GAL+	A346A
VG4162-2A	MATa smc1-T1117I scc2-4 LacO-NAT::lys4 GFPLacI-HIS3:his3-11,15 trp1-1 leu2-3,112 ura3-52 bar1 GAL+	A346A
VG4144-5C	MATa smc3Δ::HPH smc3-K112R,K113R-LEU2:leu2-3,112 GFPLacI-TRP1:his3-11,15 trp1-1 LacO-NAT::lys4 ura3-52 bar1 GAL+ w/pFC3	A346A
VG4144-5C Trp	MATa smc1-T1117W smc3Δ::HPH smc3-K112R,K113R-LEU2:leu2-3,112 GFPLacI-TRP1:his3-11,15 trp1-1 LacO-NAT::lys4 ura3-52 bar1 GAL+	A346A
VG4144-5C Phe	MATa smc1-T1117F smc3Δ::HPH smc3-K112R,K113R-LEU2:leu2-3,112 GFPLacI-TRP1:his3-11,15 trp1-1 LacO-NAT::lys4 ura3-52 bar1 GAL+	A346A
VG4144-5C Ala	MATa smc1-T1117A smc3Δ::HPH smc3-K112R,K113R-LEU2:leu2-3,112 GFPLacI-TRP1:his3-11,15 trp1-1 LacO-NAT::lys4 ura3-52 bar1 GAL+	A346A
VG4144-5C Asp	MATa smc1-T1117D smc3Δ::HPH smc3-K112R,K113R-LEU2:leu2-3,112 GFPLacI-TRP1:his3-11,15 trp1-1 LacO-NAT::lys4 ura3-52 bar1 GAL+	A346A

VG4144-5C Glu	MATa smc1-T1117E smc3Δ::HPH smc3-K112R,K113R-LEU2:leu2-3,112 GFPLacI-TRP1:his3-11,15 trp1-1 LacO-NAT::lys4 ura3-52 bar1 GAL+	A346A
VG4144-5C Gly	MATa smc1-T1117G smc3Δ::HPH smc3-K112R,K113R-LEU2:leu2-3,112 GFPLacI-TRP1:his3-11,15 trp1-1 LacO-NAT::lys4 ura3-52 bar1 GAL+	A346A
VG4144-5C Ser	MATa smc1-T1117S smc3Δ::HPH smc3-K112R,K113R-LEU2:leu2-3,112 GFPLacI-TRP1:his3-11,15 trp1-1 LacO-NAT::lys4 ura3-52 bar1 GAL+	A346A
VG4144-5C Lys	MATa smc1-T1117K smc3Δ::HPH smc3-K112R,K113R-LEU2:leu2-3,112 GFPLacI-TRP1:his3-11,15 trp1-1 LacO-NAT::lys4 ura3-52 bar1 GAL+	A346A
VG4154-3A	MATa wpl1Δ::HPH smc3-K112R,K113R LacO-NAT::lys4 GFPLacI-HIS3:his3-11,15 trp1-1 leu2-3,112 ura3-52 bar1 GAL+	A346A
VG4153-5C	MATa smc1-D1164E smc3-K112R,K113R TIR1-CgTRP1 LacO-NAT::lys4 GFPLacI-HIS3:his3-11,15 leu2-3,112 ura3-52 bar1 GAL+	A346A
VG4158-9D	MATa smc1-T1117W smc3-K112R,K113R-LEU2:leu2-3,112 smc3Δ::HPH LacO-NAT::lys4 GFPLacI-TRP1:his3-11,15 trp1-1 ura3-52 bar1 GAL+	A346A
VG3991-1A	MATa smc3-K112R,K113R-LEU2:leu2-3,112 SMC3-N607-3V5-AID TIR1-CgTRP1 LacO-NAT::lys4 GFPLacI-HIS3:his3-11,15 ura3-52 bar1 GAL+	A346A
VG4168-7B	MATa smc1-T1117W TIR1-CgTRP1 LacO-NAT::lys4 GFPLacI-HIS3:his3-11,15 leu2-3,112 ura3-52 bar1 GAL+	A346A
VG4138-1A	MATa smc1-D1164E TIR1-CgTRP1 LacO-NAT::lys4 GFPLacI-HIS3:his3-11,15 leu2-3,112 ura3-52 bar1 GAL+	A346A
VG3961-4B	MATa smc3Δ::HPH LacO-NAT::lys4 GFPLacI-TRP1:his3-11,15 trp1-1 leu2-3,112 ura3-52 bar1 GAL+ w/pFC3	A346A
VG3965-1A	MATa smc1Δ::HPH LacO-NAT::lys4 GFPLacI-HIS3:his3-11,15 trp1-1 leu2-3,112 ura3-52 bar1 GAL+ w/pFC1	A346A
KB58A	MATa pGAL1,10-GAL4, SMC1-PK3-ADE2:ade2-1, pGAL1,10-SCC3-MYC-URA3:ura3, pGAL1,10 SMC3,MCD1-3C-3xStrepII-TRP1:trp1, can1-100, leu2-3,112, his3, GAL, psi+, pep4Δ::HIS3 wpl1Δ::LEU eco1Δ::KANMX6	K699
KB140A	MATa pGAL1,10-GAL4, SMC1-PK3-ADE2:ade2-1, pGAL1,10-SCC3-MYC-URA3:ura3, pGAL1,10 smc3-K113Q, MCD1-3C-3xStrepII-TRP1:trp1, can1-100, leu2-3,112, his3, GAL, psi+, pep4Δ::HIS3 wpl1Δ::LEU eco1Δ::KANMX6	K699
SX313	MATa pGAL1,10-GAL4, SMC1-T1117I-PK3-ADE2:ade2-1, pGAL1,10-SCC3-MYC-URA3:ura3, pGAL1,10 smc3-K113Q, MCD1-	K699

	3C-3xStrepII-TRP1:trp1, can1-100, leu2-3,112, his3, GAL, psi+, pep4Δ::HIS3 wpl1Δ::LEU eco1Δ::KANMX6	
SX312	MATa pGAL1,10-GAL4, SMC1-T1117I-PK3-ADE2:ade2-1, pGAL1,10-SCC3-MYC-URA3:ura3, pGAL1,10 SMC3,MCD1-3C-3xStrepII-TRP1:trp1, can1-100, leu2-3,112, his3, GAL, psi+, pep4Δ::HIS3 wpl1Δ::LEU eco1Δ::KANMX6	K699
SX318	MATa pGAL1,10-GAL4, SMC1-T1117W-PK3-ADE2:ade2-1, pGAL1,10-SCC3-MYC-URA3:ura3, pGAL1,10 SMC3,MCD1-3C-3xStrepII-TRP1:trp1, can1-100, leu2-3,112, his3, GAL, psi+, pep4Δ::HIS3 wpl1Δ::LEU eco1Δ::KANMX6	K699
SX316	MATa pGAL1,10-GAL4, SMC1-D1164E-PK3-ADE2:ade2-1, pGAL1,10-SCC3-MYC-URA3:ura3, pGAL1,10 SMC3,MCD1-3C-3xStrepII-TRP1:trp1, can1-100, leu2-3,112, his3, GAL, psi+, pep4Δ::HIS3 wpl1Δ::LEU eco1Δ::KANMX6	K699
SX305	MATa lys2 pep4::HIS3 ade2-1::ADE2-pGAL-GAL4 trp1Δ2 leu2-3, 112 ura3-52 ade2-1 can1-100 bar1::hisG w/[2u URA3 pGAL1,10-(Scc2-MYC-Strept)-(Scc4)]	W303
VG4210-1D	MATa smc1-T1117I scc2Δ::G418 TIR1-CgTRP1 LacO-NAT::lys4 GFPLacI-HIS3:his3-11,15 leu2-3,112 ura3-52 bar1 GAL+ w/pVG587 (SCC2 URA3 CEN)	A346A
VG4215-1B	MATa smc1-T1117I smc3-K113Q scc2Δ::G418 TIR1-CgTRP1 LacO-NAT::lys4 GFPLacI-HIS3:his3-11,15 leu2-3,112 ura3-52 bar1 GAL+ w/pVG587 (SCC2 URA3 CEN)	A346A
VG4215-1B + p3555	MATa smc1-T1117I smc3-K113Q scc2Δ::G418 TIR1-CgTRP1 LacO-NAT::lys4 GFPLacI-HIS3:his3-11,15 leu2-3,112 ura3-52 bar1 GAL+ w/pVG587 (SCC2 URA3 CEN) & p3555 (SCC2 LEU2 CEN)	A346A
VG4215-1B + pRS315	MATa smc1-T1117I smc3-K113Q scc2Δ::G418 TIR1-CgTRP1 LacO-NAT::lys4 GFPLacI-HIS3:his3-11,15 leu2-3,112 ura3-52 bar1 GAL+ w/pVG587 (SCC2 URA3 CEN) & pRS315 (LEU2 CEN)	A346A
KB40A	MATa TIR1-CgTRP1 LacO-NAT::lys4 GFPLacI-HIS3:his3-11,15 leu2-3,112 pGAL-SCC2/SCC4-URA3:ura3-52 bar1 GAL+	A346A
