# Supplementary material for: A model for Scc2p stimulation of cohesin's ATPase and its inhibition by acetylation of Smc3p
Source: Genes Dev. 2023 Apr 1;37(7-8):277–90. doi: 10.1101/gad.350278.122 (PMC10153460; doi:10.1101/gad.350278.122)
Supplement: Supplemental Material [file supp_gad.350278.122_Supplemental_Table2List.docx]

Supplemental Table 2 – Plasmid Table

Integrative Plasmids

| Plasmid name | Yeast Genes |
| --- | --- |
| pVG556 | pGAL-SCC2, pGAL-SCC4 LEU2 |
| pFC5 | pGAL-SCC2 pGAL-SCC4 URA3 |
| pVG444 | SMC1 LEU2 |
| pVG444 A159T | smc1-A159T LEU2 |
| pVG444 N220K | smc1-N220K LEU2 |
| pVG444 T1117I | smc1-T1117I LEU2 |
| pVG419 | SMC3 LEU2 |
| pVG419 K113Q | smc3-K113Q LEU2 |
| pVG419 E305K | smc3-E305K LEU2 |
| pVG419 K113Q, E305K | smc3-K113Q, E305K LEU2 |
| pVG419 V492G | smc3-V492G LEU2 |
| pVG419 K113Q, V492G | smc3-K113Q, V492G LEU2 |
| pVG419 R1199L | smc3-R1199L LEU2 |
| pVG419 K113Q, R1199L | smc3-K113Q, R1199L LEU2 |

Centromere Plasmids

| Plasmid name | Yeast genes |
| --- | --- |
| pFC1 | S.bay SMC1 URA3 G418 |
| pFC3 | S.bay SMC3 URA3 G418 |

CRISPR Plasmids

| Plasmid | Guide Target | Primers | Parent plasmid |
| --- | --- | --- | --- |
| pFC8 | Smc1-T1117 | VG774/VG775 | pJR3429 |
| pVG524 | Smc1-K113 | VG727/VG728 | pVG522 |
| pFC9 | Smc1-D1164 | VG766/VG767 | pJR3429 |

Purification Plasmids

| Purification Plasmids | Yeast Genes |
| --- | --- |
| pSX103 | pGAL-SMC1-PK3, pGAL-GAL4 ADE2 |
| pSX104 | pGAL-SCC3-MYC URA3 |
| pKB11 | pGAL-SMC3, pGAL-MCD1-3C-3xStrepII TRP1 |
| pKB57 | pGAL-Smc1T1117I-PK3, pGAL-GAL4 ADE2 |
| pKB58 | pGAL-Smc1-T1117W-PK3, pGAL-GAL4 ADE2 |
| pKB47 | pGAL-Smc3-K113Q, pGAL-MCD1-3C-3xStrepII TRP1 |
| pKB49B | pGAL-Smc3-K38I, pGAL-MCD1-3C-3xStrepII TRP1 |

2-micron Plasmid

| Purification Plasmids | Yeast Genes |
| --- | --- |
| GC1206 | pGAL-SCC4, pGAL-SCC2-MYC3-3xStrepII URA3 |
